# Digital Inequalities in the Use of eHealth Services in European Public Health Care Systems: Systematic Review of Observational Studies

**DOI:** 10.2196/81841

**Published:** 2026-02-09

**Authors:** Garazi Monasterio, Marcos José Fernández-López, Erika Valero, Unai Martin, Amaia Ayala-García

**Affiliations:** 1 Department of Nursing I, Faculty of Medicine and Nursing, University of the Basque Country (UPV/EHU), Barrio Sarriena s/n, Leioa, 48940, Spain, 34 946015499; 2 Research Group on Social Determinants of Health and Demographic Change-OPIK, University of the Basque Country (UPV/EHU), Leioa, Spain; 3 Department of Sociology and Social Work, Faculty of Social Sciences and Communication, University of the Basque Country (UPV/EHU), Leioa, Spain; 4 Biobizkaia Health Research Institute, Barakaldo, Spain

**Keywords:** eHealth, digital health, health services accessibility, delivery of health care, health inequities, health care disparities, social determinants of health, public health infrastructure

## Abstract

**Background:**

European public health care systems are expanding eHealth tools such as teleconsultations, online appointment bookings, and electronic health records to improve efficiency and access to health care. However, their use depends on factors such as digital skills and internet access, which are unequally distributed across socioeconomic and demographic determinants. Most existing evidence on these inequalities is qualitative or outside universal health care systems.

**Objective:**

This systematic review aims to synthesize quantitative evidence on social inequalities in access to and use of eHealth services within European public health care systems. Specifically, we sought to identify which social determinants were most consistently associated with unequal use of online appointment booking, teleconsultations, electronic health records, and eHealth portals, across major social determinants of health.

**Methods:**

A systematic search was conducted across PubMed, Scopus, Web of Science, and PsycINFO for studies published in English or Spanish between 2015 and October 2025. Eligible quantitative studies included adults (≥18 years) using public health care systems in European countries. The primary outcome was differential access to or use of eHealth tools by social determinants in any level of care. Screening and data extraction were independently performed by 3 reviewers using Rayyan, resolving disagreements through consensus. Data extracted covered study design, population, eHealth tools, social determinants, and outcomes. Risk of bias was evaluated using Joanna Briggs Institute tools. Due to study heterogeneity in digital tools and inequality dimensions, results were synthesized narratively by tool type and social inequality factors. Point estimates and 95% CIs were extracted when available.

**Results:**

Of the 2366 records retrieved, 18 observational studies met the inclusion criteria: 13 cross-sectional, 3 prevalence, 1 retrospective cohort, and 1 ecological cohort. Publication output increased from 2020 onward, mostly driven by cross-sectional studies from northern and western Europe. Findings revealed consistent social gradients in eHealth use: older adults, individuals with lower educational or socioeconomic level, ethnic minorities, and those with limited digital skills or poorer health were less likely to use eHealth tools. Most studies were rated as high quality (78%), and the remainder as moderate, heterogeneity in designs, outcomes, and populations may limit generalizability.

**Conclusions:**

Digital transformation in European public health systems has not benefited all groups equally. This review highlights persistent social inequalities in the use of key digital health tools. While many included studies were of high quality, heterogeneity in study designs, populations, and outcomes, as well as risk of bias, limits causal inference and the direct translation of findings into policy and practice. The findings nonetheless reveal systematic patterns of exclusion that are highly relevant for policy. Emphasizing an intersectional approach and standardizing measures of digital access will be essential to develop effective, equity-focused policies that ensure inclusive digital health services for all.

## Introduction

Health systems worldwide are undergoing a strong shift toward eHealth [1,2], also referred to as digital health, telehealth, or telemedicine. This transition, long encouraged by institutions such as the World Health Organization (WHO) [3] to achieve the 2030 Sustainable Development Goals, was accelerated by the COVID-19 pandemic [4,5].

One of the earliest and most widespread applications of eHealth is the online appointment booking, teleconsultations, access to electronic health records (EHRs), and integrated digital health portals that bundle multiple services in a single interface [6,7]. These 4 modalities constitute the most common digital interaction points with health care services across European public health systems and form the conceptual foundation for evaluating digital health access and usage in this review [8]. Beyond offering advantages for health care systems and for patients [9,10], these tools have also been identified as potential levers for improving access in underserved settings, such as rural or remote areas [11].

However, there is comparatively less research on how these same tools may reinforce or even exacerbate existing social inequalities in access and usage. As Western et al [12] have conceptualized, these inequalities manifest across 3 interrelated levels of the digital divide: (1) access to internet connectivity and digital devices; (2) digital skills and literacy, trust in technology, and willingness to adopt new platforms; and (3) disparities in actual outcomes or health improvements derived from digital tool use. Only those who overcome these first 2 levels can potentially benefit, leading to the third digital health divide. Breakdowns at any of these levels can cascade to reinforce exclusion at the next, perpetuating digital and health inequities. Quantitative studies, many outside universal health care settings, point out that older adults, people with lower socioeconomic status or education [13], migrated population, ethnic minorities, rural population, or in low-skilled jobs [14], individuals with impairments [15], and other socially disadvantaged groups often lack these prerequisites [16], revealing measurable gaps in digital health use [17,18]. Consequently, digital health strategies may risk amplifying pre-existing health disparities [19]. In addition, digital divide measurement methods and indicators vary widely, from composite indexes (aggregated scores combining multiple dimensions of access and use) to simple usage metrics (such as number of logins or appointments booked) [18,20]. Yet, the absence of harmonized indicators makes it difficult to compare findings across countries, especially in health care systems with very different structures.

Within Europe’s public health care systems—across all levels of care—there remains scant data quantifying inequalities in basic digital functions such as booking appointments, accessing health portals, or having teleconsultations.

Despite these concerns, public health systems across Europe (and beyond) are investing heavily in digital care models [21]. While this expansion signals progress, it also highlights the lack of robust monitoring of equity impacts. Most existing studies emphasize qualitative evidence, which provides valuable perspectives but limited measurement of scale [22]. Quantitative research is less common, and when available, it rarely disaggregates results by key social determinants, leaving important gaps in understanding how digital health inequalities unfold across vulnerable groups.

In recent years, this issue has attracted renewed policy and research attention, reflecting a growing recognition that digital transformation alone does not guarantee equitable access [23]. Several recent analyses and policy reports have documented substantial advances in national eHealth strategies and portal deployment across Europe, while noting that evaluation frameworks for equity and inclusion remain limited [24,25]. At the same time, comparative assessments by international organizations emphasize the need for harmonized indicators and monitoring systems to assess how digitalization affects different population groups within public health care [26]. This evolving evidence base reinforces the importance of systematically consolidating quantitative findings to clarify where inequalities persist and how they are measured within European public health systems.

In this context and considering that the WHO’s Global Strategy on Digital Health 2020‐2025 (extended to 2027) emphasizes that eHealth should enhance universal access, quality, efficiency, and equity [8], there is an urgent need to identify who is falling behind. To date, no systematic review has synthesized quantitative evidence on these digital inequalities across European public health care systems.

The objective of this systematic review is, therefore, to synthesize the available quantitative evidence on inequalities in access to and use of core eHealth services—including online appointment booking, teleconsultations, and EHRs—within European public health care systems between 2015 and 2025, examining how key social determinants, such as age, gender, socioeconomic status, education, migration background, and digital literacy, are associated with the use of these services.

## Methods

### Overview

A systematic review was conducted to identify studies examining inequalities in the use of eHealth technologies to access health care, provided by public health care systems, in relation to social determinants of health in Europe. This review was carried out in accordance with the PRISMA (Preferred Reporting Items for Systematic Reviews and Meta-Analyses) [27] guidelines to ensure a rigorous and transparent evaluation of the review process. For details of items included in the PRISMA checklist, please see Checklist 1. The protocol was registered in the PROSPERO database (registration number: CRD420251015756).

### Information Sources and Search Strategy

A systematic search was performed in PubMed, Scopus, PsycINFO, and Web of Science up to April 2025. The search strategy combined controlled vocabulary and free-text terms related to digital health (eg, “telemedicine,” “eHealth,” “digital health,” and “patient portal”), social inequalities (eg, “health disparities,” “digital divide,” “inequality,” and “equity”), social determinants and user characteristics (eg, “age,” “gender,” “ethnic minorities,” “disability,” and “socioeconomic”), health care access and use (eg, “access,” “use,” “user,” and “nonuser”) and geographical terms referring to Europe and individual European countries (eg, “Europe,” “United Kingdom,” “Germany,” and “Spain”). Truncation and Boolean operators (AND, OR, NOT) were used to combine these concepts and optimize sensitivity and specificity. The search strategy was developed collaboratively by the research team, without consultation with an information scientist. Complete search strategies and term lists are available in the Multimedia Appendix 1.

The search process and its reporting followed the PRISMA-S (Preferred Reporting Items for Systematic Reviews and Meta-Analyses literature search extension) guidelines to ensure transparency, completeness, and reproducibility of each search component [28]. In addition, a backward citation search (snowballing) was conducted from the reference lists of the included studies. The literature search was rerun on October 20, 2025, to capture any recent publications since the initial search.

### Inclusion and Exclusion Criteria

Studies were eligible for inclusion if they met the following criteria: (1) study population focused on adults aged 18 years or older. Studies that included younger populations but allowed for interpretation of results in adults more than 18 years were also included; (2) assessed any type of eHealth technology designed to connect individuals to public health systems; (3) reported quantitative data related to use of digital health services provided by public health systems in association with social determinants of health; (4) were conducted in European countries, defined as member states of the European Union, the European Economic Area (Iceland, Liechtenstein, and Norway), the United Kingdom, and Switzerland. This definition was selected to ensure conceptual and structural comparability of health care systems with public health care systems coverage and established digital health infrastructure; and (5) were peer-reviewed studies published in English or Spanish between January 2015 and April 2025.

The following exclusion criteria were applied: (1) studies focusing exclusively on populations using private health care services; (2) focusing on the effects of the COVID-19 pandemic on eHealth usage; (3) focusing on health apps designed for preventive purposes, such as interventions, general health information provision, self-care, or supporting the management of specific conditions; and (4) qualitative studies, study protocols, conference abstracts, theses, editorials, opinion pieces, and systematic reviews.

### Data Management and Study Selection

Study screening was conducted using the Rayyan web-based tool [29]. No automation tools were used in this process. Two researchers independently screened titles and abstracts (GM and AA), followed by full-text review of potentially eligible studies. Disagreements were resolved through a third reviewer (MF). Study authors were contacted when additional information or clarification was required.

### Data Extraction

Data extraction was carried out collaboratively by 3 researchers (GM, AA, and MF) and reviewed by the other team members to ensure accuracy and consistency. The following information was extracted: authorship, year of publication, country, study design, population characteristics, type of eHealth technology, outcome, social determinants of health included, and key quantitative findings and their narrative description. In addition, eHealth tools identified in the studies were regrouped into 4 categories according to their functionality in (1) eHealth portals—which typically integrate multiple functionalities into a single tool. These include services such as prescription renewal, appointment reminders, asynchronous messaging with health care professionals, access to test results, and in some cases, teleconsultation features or visualization of EHRs; (2) EHRs—this category includes those tools that allow users to consult their medical results, diagnoses, clinical notes, and other information recorded in their health record. These functionalities may be, and often are, integrated into digital portals managed by public health services or specific hospital apps. Notable examples include platforms such as MyCare, myHealth@QEHB, or the Care Information Exchange in the United Kingdom; (3) telemedicine and remote primary care—understood as the provision of direct health care through digital channels. It includes video medical consultations, direct-to-consumer digital care models, as well as the remote provision of primary care; (4) online appointment booking. This classification enabled a more nuanced analysis of inequalities based on the specific functionality assessed in each study. To ensure clarity in the tables, this categorization is represented numerically (1-4).

Given the variability in terminology and definitions across included studies, we compiled a comparative table showing how each study defined key determinants and eHealth usage, which is provided in Multimedia Appendix 2.

### Risk of Bias Assessment

Risk of bias was assessed using the appropriate critical appraisal tools developed by the Joanna Briggs Institute (JBI), selected based on each study’s methodological design (eg, cross-sectional and cohort) [30]. The assessment was conducted at the study level rather than at the outcome level. This approach was chosen because the included studies were primarily observational and reported heterogeneous outcomes, making study-level assessment the most consistent and feasible strategy. The appraisal focused on key domains affecting internal validity, including participant selection, measurement of exposures and outcomes, and control of confounding factors. Studies were classified into 3 quality categories based on the proportion of affirmative responses to the JBI appraisal items: high quality (≥70%), moderate quality (40%‐69%), and low quality (≤39%) [31]. A detailed summary of the risk of bias assessment is provided in Multimedia Appendix 3 [32-49], using the corresponding JBI Critical Appraisal Tool.

At least 2 reviewers assessed the risk of bias independently. Discrepancies were resolved through discussion and consensus, involving 3 researchers (GM, AA, and MF) during this phase.

### Data Synthesis and Effect Measures

The effect measures reported in the studies varied. Some studies presented descriptive comparisons between population subgroups (such as percentages or proportions), while others reported adjusted measures of association derived from regression models, such as odds ratios (OR) with corresponding CIs. When available, information was classified according to the type of eHealth service analyzed.

In addition, some studies applied more advanced equity metrics, such as the concentration index and the horizontal inequity index. The concentration index summarizes whether use of services is disproportionately concentrated among socioeconomically advantaged or disadvantaged groups. The horizontal inequity index adjusts for differences in health care needs (eg, morbidity), isolating inequities that persist beyond expected variations in medical necessity. A few studies also used decomposition analysis to examine the contribution of individual determinants (eg, education, income, and employment) to overall inequality, and indirect standardization to account for differences in health needs when comparing groups.

Due to the heterogeneity in study designs, eHealth technologies evaluated, and inequality dimensions analyzed, a narrative synthesis was therefore conducted to summarize the findings. Results were presented in evidence tables and described narratively in the text. Studies with comparable outcomes were grouped, as explained in the “Data Extraction” section.

## Results

### Study Selection

**Figure 1.**
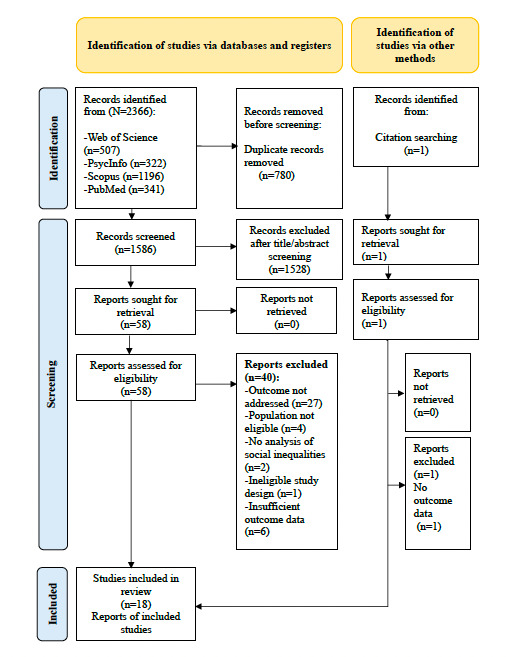
PRISMA (Preferred Reporting Items for Systematic Reviews and Meta-Analyses) 2020 flow diagram of study selection process.

The initial search identified 2366 records from 4 databases. Of these, ultimately, 18 studies [32-49] were included in the systematic review. A PRISMA 2020 flow diagram detailing the selection process is presented in Figure 1.

### Characteristics of the Studies Included

**Figure 2.**
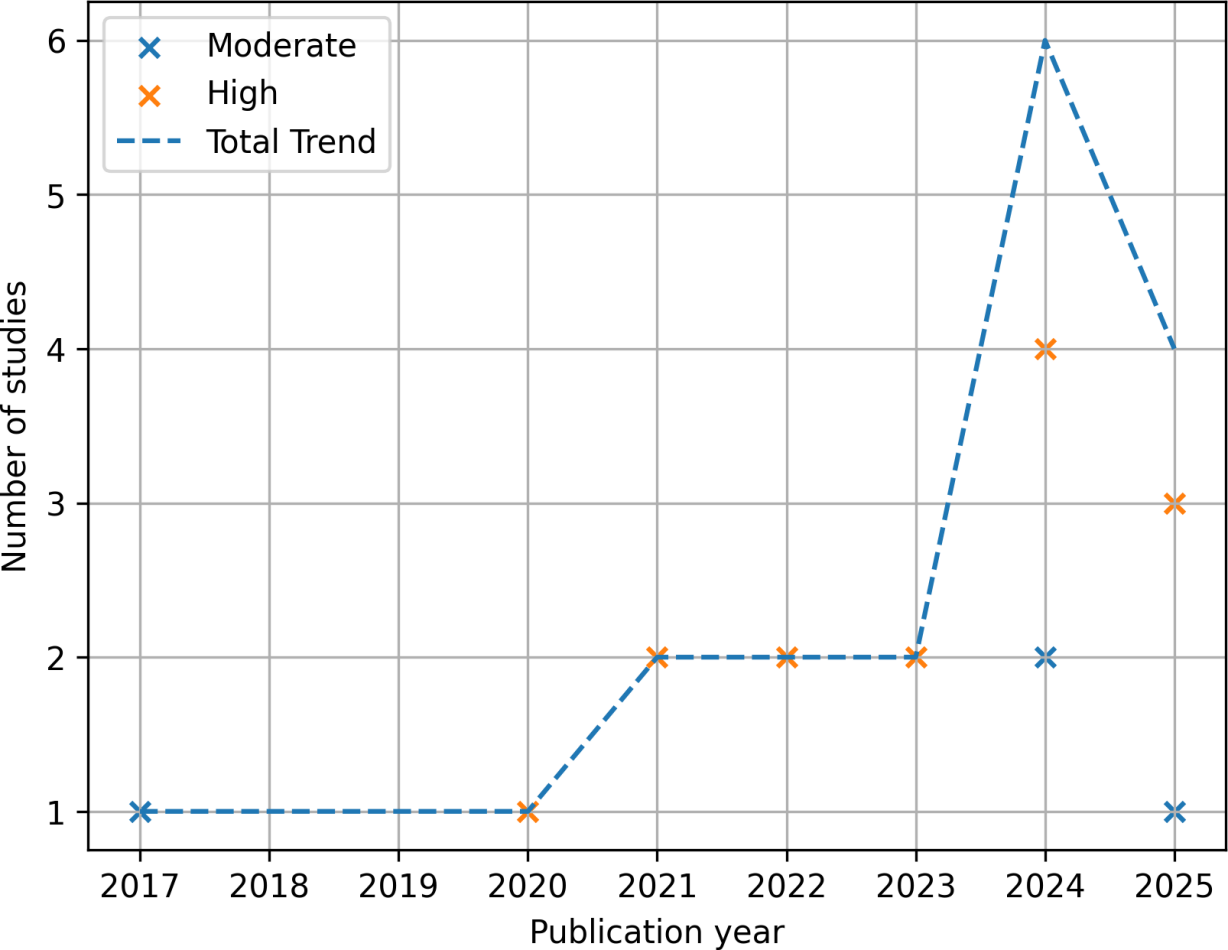
Publications per year by quality category.

The included studies were published between 2017 and 2025. A general upward trend in the number of publications was observed over time, with a noticeable increase in high-quality studies in the most recent years. The distribution of included studies per year according to their methodological quality category is illustrated in Figure 2, where the trend line represents the total number of studies published per year, and the green and yellow “x”’s represent the quality of the individual studies per year.

The most frequent study design was cross-sectional observational, accounting for 72% (13/18) of the studies [32-37,39-42,45,46,49], followed by prevalence studies at 17% (3/18) [38,43,47], with one retrospective cohort [44] and one ecological cohort study each representing 6% (1/18) [48]. The 18 studies [32-49] were conducted across 10 European countries, with the majority from Northern and Western Europe. Sweden had the highest number of studies, representing 28% (5/18) [33,36,37,44,45], followed by England at 22% (4/18) [35,46,48,49] and Finland at 11% (2/18) [32,41]. Other countries contributed one study each: Spain [42], Norway [47], Germany [40], Iceland [38], Denmark [43], the United Kingdom (distinct from England) [34], and one pan-European study [39]. Sample sizes ranged from under 438 to 1,991,995 participants. They varied from small studies such as Hörhammer et al [32] in Finland (n=438) to very large population-based datasets such as Dahlgren et al [33] in Sweden (n=1,991,995). Specifically, 17% (3/18) of studies had sample sizes below 1000 [32,34,35], 44% (8/18) ranged between 1000 and 10,000 [36-43], 28% (5/18) exceeded 10,000 participants [33,44-47], and 11% (2/18) did not report sample size [48,49]. Most studies focused on the general adult population aged 18 years and older.

### Risk of Bias Assessment

According to the risk of bias assessment, 22% (4/18) of the studies [40,42,43,48] were classified as of moderate quality, while the remaining 78% (14/18) were rated as high quality [32-39,41,44-47,49]. No studies were considered to have low methodological quality, suggesting that the overall quality of the evidence included was generally high.

**Figure 3.**
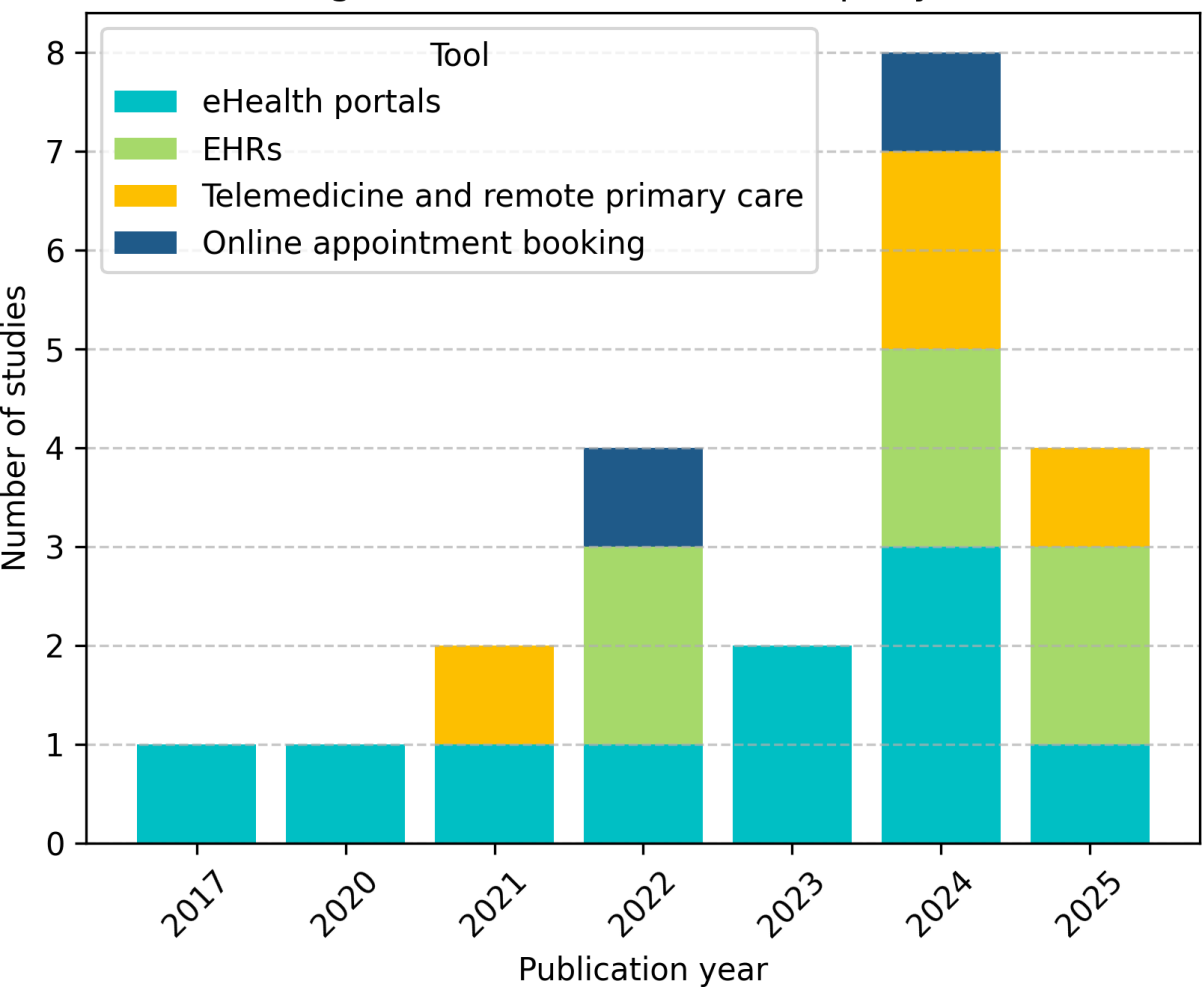
Assessment of eHealth tools per year. EHR: electronic health record.

The eHealth tools analyzed were regrouped into 4 functional categories as described in the “Methods” section: (1) eHealth portals, (2) EHRs, (3) telemedicine and remote primary care, and (4) online appointment booking. Of the 18 included studies [32-49], 56% (10/18) analyzed eHealth portals [32,34,35,38-41,43,48,49], 33% (6/18) examined EHRs [37,41,42,46-48], 22% (4/18) addressed telemedicine and remote primary care [33,36,44,45], and only 11.1% (2/18) focused on online appointment booking [41,48]. Figure 3 presents the distribution of eHealth tool categories by publication year, highlighting a growing diversification in digital health functionalities over time. Studies appear more than once when assessing more than one tool usage.

**Figure 4.**
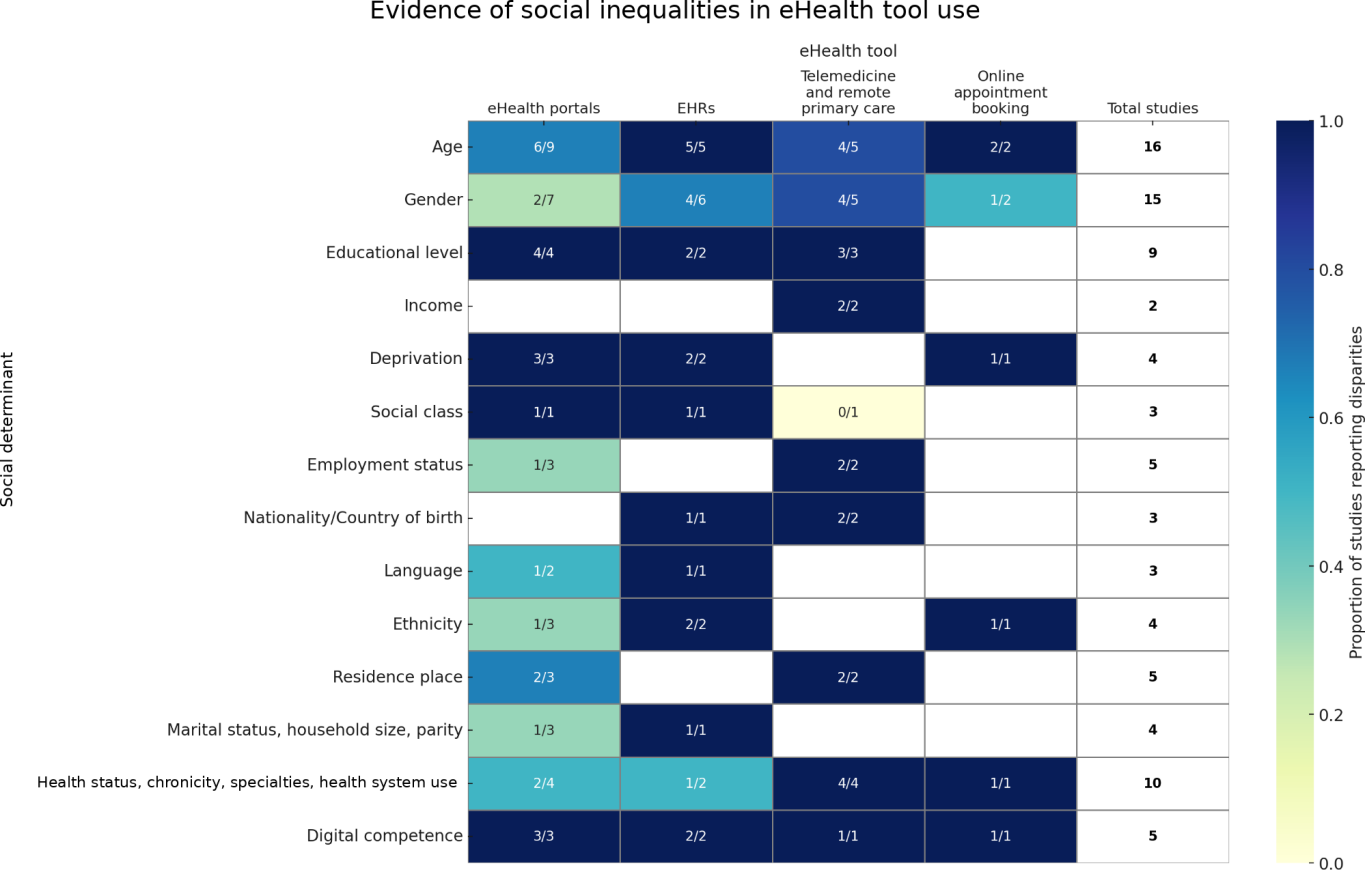
Evidence of social inequalities in eHealth tool use. EHR: electronic health record.

All 18 studies reviewed examined social inequalities in the use of eHealth tools, with age and gender being the most analyzed determinants [32-49]. A second major block of variables related to socioeconomic position, especially social class [39,43,44], education [33,34,36,37,42,45], employment [32,36,40,45], income [33,45], and deprivation [35,46,48,49], often using both individual-level and area-based indicators. Fewer studies addressed cultural-related factors such as ethnicity [34,46,48,49], country of birth [33,45], nationality [42], and language [35,40,46], though these were sometimes used as proxies for integration and accessibility. Determinants related to residential context (eg, urban vs rural [40,45,49] and distance to care [33]) and family structure (eg, marital status [37,39,40], household size [39], and parity [35]) were included less frequently. Health status and clinical complexity were operationalized in diverse ways, including self-rated health [34,36,37], chronic conditions [32,33,48,49], diagnoses or number of specialties involved [45,46] or resource use band [44]. Finally, several studies assessed digital competence [41,42], literacy [32,34], and internet habits [36], highlighting the role of skills and confidence in shaping access to digital health care. Figure 4 presents a heatmap summarizing the extent to which each social determinant was not only examined but also found to be associated with inequalities in the use of specific eHealth tools. Each cell displays the proportion of studies that reported significant disparities out of those that analyzed a given determinant for each tool. For example, a value of “5/5” under “gender” and “EHRs” indicates that all 5 studies assessing gender differences in the use of EHRs reported significant inequalities by this determinant. This visual summary highlights both the breadth of research on each determinant and the consistency of evidence across different tools. eHealth portals were the most frequently studied, covering a wide range of social factors; however, the findings were somewhat mixed, with inequalities observed in some areas (eg, age and education) but not others (eg, gender and ethnicity). In contrast, studies on EHRs and telemedicine and remote primary care, though less numerous, tended to yield more consistent evidence of social inequalities, particularly along the lines of age, gender, and socioeconomic status. Finally, online appointment booking was the least explored modality, with very limited evidence available.

A summary of the main characteristics and key findings of the included studies is presented in Table 1. A more detailed version is available in Multimedia Appendix 4 [32-49].

**Table 1.** Evidence table summarizing the main information of each study included in the systematic review, ordered by study design.

Author (year); Country	Design, Sample, Population, and Statistical analysis	eHealth Cat^a^ and study Outcome	SDH^b^	Description of main finding of inequalities in the studies
González-Cacheda et al [42] (2025); Spain	Cross-sectional Population present in the Health Barometer 2022 (n=7454) Univariate logistic regressions	(2) Use of medical records via internet	Age Gender Education Nationality Social class Device use	Older adults: higher awareness (OR^c^ 0.86) but lower effective use than younger and middle-aged. No gender differences. Higher education (OR 1.41) and digital experience (OR 1.51) associated with greater use. Spanish nationals are more likely to use digital medical records (OR 1.45). 95% CI not reported in the primary study.
Hörhammer et al [32] (2025); Finland	Cross-sectional Patients from a Finnish mental health and substance abuse unit (n=438) Univariate and multivariate logistic regressions	(1) Use of digital services	Age Gender Health confidence Employment Duration of care	Older adults (≥65 years; OR 0.40, 95% CI 0.21‐0.75) less likely to use digital services. Low health confidence is associated with reduced use (OR 0.62, 95% CI 0.45‐0.86).
Knöchelmann et al [40] (2024); Germany	Cross-sectional Adults (≥ 18 years) from the HeReCa panel (n=1821) Descriptive analysis, latent class analysis, and multinomial logistic regression	(1) Use of digitalized health care services	Age Gender Education Language Employment Place of residence Marital status	No significant associations for women (OR 0.78, 95% CI 0.56‐1.10), age (OR 0.88, 95% CI 0.71‐1.08), or marital status (married vs others; OR 0.77, 95% CI 0.53‐1.13). Higher education is linked to greater odds of rejecting participation (OR 1.23) but lower odds of active use (OR 0.68). 95% CI not reported in the primary study. Lower educational attainment (OR 0.67, 95% CI 0.47‐0.95) and unemployment (OR 0.57, 95% CI 0.38‐0.84) associated with reduced active use. Number of previous illnesses not associated with use.
Söderberg et al [36]; Sweden	Cross-sectional Adults (≥ 18 years) residing in Sweden (n=2716) Multivariate logistic regression	(3) Use of digital primary care	Age Gender Occupation Education Internet habits Self-rated health	Younger age associated with higher likelihood of seeking digital primary care (95% CI not reported in the primary study). University education (OR 1.41, 95% CI 1.19‐1.67) and daily internet use (OR 3.21, 95% CI 1.30‐7.90) linked to greater use. Retired individuals had lower odds than those working (OR 0.68, 95% CI 0.46‐1.02; borderline nonsignificant).
Wilkens et al (2024) [45]; Sweden	Cross-sectional Patients ≥18 years (n=726,087) Concentration index and curves; decomposition analysis; indirect standardization; horizontal inequity index	(3) Use of digital primary care	Age Gender Education Morbidity Country of birth Geographic region Employment	Digital care users were younger and more often women. Low-income patients used more office-based visits, whereas high-income patients had more digital contacts; income and employment explained most inequalities in office visits. Pro-rich inequality in digital contacts was partly explained by higher education and being born in Sweden; digital users were generally healthier.
Muli et al (2024) [37]; Sweden	Cross-sectional Patients ≥18 years and guardians of minors living in Region Stockholm who had a consultation with a physician (n=3421) Multivariate logistic regressions	(2) Access to patient-accessible electronic health records	Age Gender Education Marital status Health status	Younger age associated with higher likelihood of reading records (OR 0.97, 95% CI 0.95‐0.98). Women are more likely to have read their electronic health records (77% readers overall). Partnered individuals (vs single; OR 0.60, 95% CI 0.44‐0.99) and those with higher education were more likely to be readers. No differences were observed by health status.
Zhang et al [49] (2023); England	Cross-sectional Populations of primary care practices in National Health Service (no n reported) Multivariate linear regression	(1) Use of National Health Service (NHS) app (NHS Digital) and a primary care portal	Age Deprivation Ethnicity Residence place Long-term condition	Practices with more male or chronically ill patients had lower usage (*P*<.01). Strong socioeconomic gradient: all more deprived quintiles showed lower digital health use than the least deprived (Q1; difference = –2.047 units; *P*<.001). Higher use in practices with more White and younger patients (15‐34 years; +10.39% registration, *P*<.05) and in larger practices (*P*<.05).
Pierce et al [35] (2023); England	Cross-sectional Pregnant women booked into UCLH^d^ for their initial antenatal appointment in February 2022, identified through the EPIC platform (n=636) Descriptive	(1) Use of MyCare, an electronic patient portal	Age Parity Language Deprivation	Non-users of MyCare were younger (mean age 30) and showed lower engagement with higher parity (mean parity =0.94, 1.47, 1.78 for high-, low-, and nonusers). Women whose first language was not English were more common among non-users (48.7%), who also had lower socioeconomic status (mean SDI^e^=3.72).
Heponiemi et al [41] (2022); Finland	Cross-sectional Sample from the Population Register of Finland >20 years (n=4495) Multivariate logistic regression	(1,2, and 4) Use of online health services	Age Gender Digital competence	Use of all digital services declined sharply after age 60, except for appointment booking. Women used online services 8% more than men. Good digital competence strongly increased use: test results (OR 12.61, 95% CI 8.52‐18.64), prescription renewal (OR 8.82, 95% CI 6.15‐12.64), appointment scheduling (OR 10.91, 95% CI 7.24‐16.44), and online appointments (OR 6.48, 95% CI 0.93‐45.12; not significant).
Chapman et al [46] (2022); England	Cross-sectional Adults ≥18 years receiving hospital outpatient care (n=28,637) Descriptive, univariate, and multivariate logistic regression	(2) Sign up and activation of myHealth @QEHB, a hospital-based Personal Health Record	Age Gender Deprivation Ethnicity Interpreted need Number of hospital specialties	Activation highest among adults aged 35‐54 years; lower for ages 16‐34 years (OR 0.80, 95% CI 0.70‐0.91) and lowest for ≥75 years (OR 0.39, 95% CI 0.32‐0.47). Males are less likely to activate their Personal Health Record (OR 0.85, 95% CI 0.78‐0.94) but more likely to sign up (OR 1.10, 95% CI 1.04‐1.16); activation is 3 times higher in the least versus the most deprived areas (OR 2.99, 95% CI 2.40‐3.71). Asian (aOR^f^ 0.61, 95% CI 0.53‐0.71), Black (aOR 0.45, 95% CI 0.36‐0.56), and mixed ethnic groups (aOR 0.77, 95% CI 0.60‐0.97) less likely to activate than White patients; those not needing an interpreter had higher activation (OR 3.16, 95% CI 1.96‐5.09). Number of clinical specialties associated with sign-up (aOR 2.54, 95% CI 2.30‐2.82) but not activation (aOR 0.88, 95% CI 0.79‐0.97).
Neves et al [34] (2021); United Kingdom	Cross-sectional Patients >18 years of hospitals and primary care in London, registered in the Care Information Exchange (n=650) Descriptive, univariate, and multivariate logistic regression	(1) Use of Care Information Exchange, a patient portal containing patient information	Age Gender Education Ethnicity Health status eHEALS^g^ score	Higher education associated with increased odds of use: undergraduate and professional (aOR 1.58, 95% CI 1.04‐2.39) and postgraduate and higher (aOR 2.38, 95% CI 1.42‐4.02). Greater digital literacy (>30) strongly predicted use (aOR 2.96, 95% CI 2.02‐4.35). Good health status linked to lower odds of use (aOR 0.58, 95% CI 0.37‐0.91).
Dahlgren et al [33] (2021); Sweden	Cross-sectional Adults (≥18 years) residing in Stockholm County and registered with a publicly funded Primary Health Care provider (n=1,991,995) Descriptive and multivariate logistic regression	(3) Use of telemedicine to provide traditional primary care	Age Gender Education Income Country of birth Diagnosis of chronic conditions Primary health care accessibility Distance to primary health care	Greater telemedicine use among women (OR 1.60, 95% CI 1.58‐1.62), younger adults (19–25), those born in Sweden, and individuals with higher education and income. Lower use among older adults (≥65 years), those born outside the EU28, with lower education or income, or with heart failure and diabetes; depression and COPD^h^ or asthma predicted higher telemedicine use. Digital care use is more concentrated in urban areas (67.5% of users).
Merkel et al [39] (2020); Europe	Cross-sectional Adults aged >65 years who use the internet (n=6900) Multilevel logistic regression	(1) Use of internet-based health care services (any service, not specified)	Age Gender Education Social class Employment Marital status Household size Population density	eHealth users were younger, better educated, and of higher social class. Older age was associated with lower use (OR 0.97, 95% CI 0.96‐0.98; *P*<.001) Higher education (16‐19 years: OR 1.43, 95% CI 1.15‐2.79; ≥20 years: OR 1.95, 95% CI 1.54‐2.46; *P*<.001) and higher social class (medium: OR 1.45, 95% CI 1.23‐1.71; high: OR 2.00, 95% CI 1.53‐2.61; *P*<.001) predicted greater eHealth use. Living with a partner increased, and living alone reduced, eHealth use. Urban residence (OR 1.23, 95% CI 1.02‐1.48; *P*=.03) and higher national education participation among older adults (OR 1.06, 95% CI 1.01‐1.13; *P*=.02) were positively associated with use.
Kharko et al [47] (2025); Norway Sweden Finland	Prevalence NORDeHEALTH 2022 Survey (n=27,038) Descriptive	(2) Access to EHR^i^	Gender	Women less likely to have never accessed the EHR or to be first-time users. Women are more likely to have visited the EHR >20 times (*χ*²_4_=57; *P*<.001).
Pálsdóttir et al [38] ; Iceland	Prevalence Adults (≥ 18 years) Residing in Iceland (n=3000) Descriptive	(1) Use of national digital health care system for communication with health professionals or access personal health information	Age Gender	Women aged 18‐35 years (*P*<.001) and 36‐55 years (*P*<.10) used the digital system more frequently. Women >56 years showed lower use compared to younger women.
Petersen et al [43] (2017); Denmark	Prevalence Danish adults (n=1059) Descriptive	(1) Access and use of Sundhed.dk, a national health portal	Education level	Individuals with only primary education were less likely to use the Danish National Health Portal than those with higher education. Portal use was 21% among those with primary education versus 60% among higher-educated individuals.
Eriksson et al [44] (2025); Sweden	Retrospective cohort (registry-based) Individuals of any age registered with Primary Health Care Centers, and having made at least 1 outpatient consultation during the study period (n=73,486) Multivariable Logistic Regression	(3) Use of telemedicine consultation in primary health care and EHR	Age	Younger adults (20-39 years) more likely to use telemedicine; older adults (60‐79 and ≥80 years) less likely (IRR^j^ 0.19, 95% CI 0.16‐0.25). Women showed higher telemedicine use (IRR 1.39, 95% CI 1.28‐1.51). Patients with high resource use (RUB 5; IRR^e^ 2.67, 95% CI 0.20‐3.54) and more EHR entries (IRR 1.50, 95% CI 1.32‐1.72) showed higher use; no significant differences by Care Need Index.
Kc et al [48] (2024); England	Ecological cohort GP-registered^k^ patients in practices with >200 patients between March 23, 2020, and June 27, 2022, aged ≥15, (no n reported) Univariate negative binomial regressions	(1, 2, and 4) Use of the NHS app for registrations, log-ins, appointments booked, prescriptions, medical record views	Age Gender Ethnicity Deprivation Health status	Higher use in practices with more patients aged 15‐34 years (+10.39% registration). Lower use across more deprived areas (up to 38.84% lower registration in most vs least deprived quintile). Use higher in larger practices and those with more White patients; lower where more male or chronically ill patients were registered.

a Author-defined grouping of eHealth tools categories.

b Social determinants of health assessed.

c OR: odds ratio.

d UCLH: University College London Hospitals.

e SDI: Social Deprivation Index.

f aOR: adjusted odds ratio.

g eHEALS: eHealth Literacy Scale.

h COPD: chronic obstructive pulmonary disease.

i EHR: electronic health record.

j IRR: incidence rate ratio.

k GP: general practitioner.

### Overall Summary

The included studies revealed consistent social inequalities in access to telemedicine, structured along key social determinants of health and disproportionately affecting vulnerable groups.

#### Age

Age was the most consistently associated determinant, with lower use among adults aged 60 years and older, and higher engagement among younger and middle-aged [32,33,35-39,41,44-46,48,49].

#### Gender

Findings regarding gender were more heterogeneous. Several studies showed lower engagement among men across different modalities [32,33,37-39,44-48], while others found no significant differences [32,34,36,39,40].

#### Socioeconomic

Lower education was strongly associated with reduced use across most studies [33,34,36,37,39,42,43,45]. Similar patterns were seen for income [33,45], social class [39,42], and area deprivation [35,46,48,49]. Unemployment [36,45] was generally linked to reduced use, though findings were mixed in some contexts [32,39,40].

#### Migration, Ethnicity, and Language

Individuals born outside the country of study [33,42,45], belonging to ethnic minorities [46,48,49], or requiring interpreter support [35,46] were less likely to access digital health care services.

#### Place of Residence

In general, people living in rural or remote areas showed lower usage levels [33,45,49] compared to those in urban or more densely populated settings. However, some studies showed mixed or context-dependent patterns [39,40].

#### Household Composition

Results were less consistent. Some studies found lower engagement among individuals without a partner [37] or with more children [35], while others reported no significant associations [39,40].

#### Health Status

The findings were mixed. Chronic illness or poor self-rated health sometimes reduced engagement [33,36,45,48,49], while others observed higher usage among those with more frequent contact with the health care system [44].

#### Digital Skills

Across the studies that assessed this dimension, lower levels of digital competence [41], limited prior digital experience [42], lack of access to a device [36], low health literacy [34], and low confidence in using technology [32] were all associated with significantly lower use of digital health services.

In the next section, we break down these findings by type of eHealth service used.

### eHealth Portal

Among the 10 studies that analyzed eHealth portals [32,34,35,38-41,43,48,49], 9 included age as a determinant [32,34,35,38-41,48,49]. Of these, 78% (6/9) found age to be a consistent factor associated with portal use [32,35,38,39,41,49]. Younger adults (15-34 years) showed higher use, and use declined after the age of 60 years [45]. Two other studies found no differences [34,46].

Regarding gender, 70% (7/10) of the studies analyzing eHealth portals included this variable [32,34,38-41,48]. Findings were mixed. Some studies found no significant differences [32,34,39,40], while others reported higher usage among women [48], and greater overall use by women [38].

Regarding socioeconomic factors, 70% (7/10) of the studies [34,35,39,40,43,48,49] examined associations between portal use and indicators such as education, income, social class, employment, and area-based deprivation. Higher education [39,40,42,45] and less deprivation [38,41,49] were consistently associated with greater use (eg, postgraduate adjusted odds ratio [aOR] 2.38, 95% CI 1.42‐4.02) [40].

Employment status was analyzed in 30% (3/10) of studies, showing inconsistent results [32,39,40]: only one study found that being used was related to lower use (OR 0.57, 95% CI 0.38‐0.84) [45]. The other two reported no associations [32,39].

Household composition, including size of household and marital status, was found as not associated in 2 of the 3 studies that analyzed it (2/10) [39,40]. However, Pierce et al [35] tied higher parity to lower use.

Cultural background—analyzed in 5 [34,35,40,48,49] of the 10 portal-focused studies (50%) [32,34,35,38-41,43,48,49]—showed no strong effects, with no significant differences in 2 studies [34,40]. Pierce et al [35] reported that 48.7% of nonusers were non-native compared to 24.4 % of those with high engagement; Kc et al [48] observed higher use in White patients [48]; Zhang [49] noted higher Asian patient use postadjustment.

In terms of area of residence, 2 [39,49] of the 3 (3/10) studies [39,40,49] that analyzed it found higher use among urban areas (OR 1.23, 95% CI 1.02‐1.48) [39] and activation [49]. Knöchelmann et al [40] found no differences.

Health status was examined in 40% (4/10) of the studies [32,34,48,49], with mixed associations: some studies reported lower use among individuals with long-term conditions [48,49], while others found higher use among those with poor health [34] or no significant differences [32].

Finally, digital competence was analyzed in 30% (3/10) of the studies, where it was described as a strong predictor [32,34,41]: (OR 8.82, 95% CI 6.15‐12.64) [41] and (aOR 2.96, 95% CI 2.02‐4.35) for high literacy [34]. Lower health literacy was associated with lower use [32].

### Electronic Health Records (EHRs)

Among the 33% (6/18) of studies [37,41,42,46-48] that analyzed EHR use, age was examined in 5, and all of them found that younger adults were more likely to use these tools [37,41,42,46,48]. Engagement declined in older adults, especially ≥75 years, with reduced registration (aOR 0.40, 95% CI 0.36‐0.44) and activation (aOR 0.39, 95% CI 0.32‐0.47) [46].

Gender was assessed in all 6 studies [37,41,42,46-48]. Four (67%) found higher EHR use among women [37,41,46-48]. In contrast, England reported higher male registration into the tool (aOR 1.10, 95% CI 1.04‐1.16) but lower activation of it (aOR 0.85, 95% CI 0.78‐0.94) [46], which was necessary for usage. González-Cacheda et al [42] found no differences.

Socioeconomic status was examined in 67% (4/6) of the studies [37,42,46,48]. All confirmed that higher levels of education increased use [37,42], and deprivation showed a clear gradient: individuals in more affluent areas were more likely to register and activate the tool [46,48].

Cultural background, assessed in 50% (3/6) of the studies [42,46,48], predicted lower activation in Asian (aOR 0.61, 95% CI 0.53‐0.71), Black (aOR 0.45, 95% CI 0.36‐0.56), and mixed (aOR 0.77, 95% CI 0.60‐0.97) groups compared to White [46]. Evidence comes primarily from a large cross-sectional study conducted in England (n=28,637) [46], complemented by national datasets from England [48] and Spain [42]. Language barriers reduced use as no interpreter need increased registration (aOR 1.63, 95% CI 1.33‐1.99) and activation (aOR 3.16, 95% CI 1.96‐5.09) [46]. National [42] and White patients [48] had higher use.

Only one study (17%, 1/6) [37], a Swedish survey of over 3000 adults, analyzed marital status, reporting that being in a partnership was linked to higher engagement [37].

Health status findings showed mixed results among the 50% of studies (3/6): one study found no differences [37], another observed lower use in long-term conditions [48], while Chapman reported higher registration among patients with complex conditions (aOR 2.54, 95% CI 2.30‐2.82) [46].

Finally, both studies assessing digital competence were conducted in Finland (n=4495) [41] and Spain (n=7454) [42], using large national datasets, and both found that lower digital skills were associated with reduced use of EHRs [41,42].

### Telemedicine and Remote Primary Care

Among the 22% (4/18) of studies [33,36,44,45] that analyzed telemedicine and remote primary care, 100% (4/4) examined age and consistently identified younger adults as the main users, while older adults showed lower use [33,36,44,45]. In Sweden, those aged 60‐79 years had substantially lower use (IRR [incidence rate ratio [0.45], 95% CI 0.40‐0.50) and those ≥80 years even lower (IRR 0.19, 95% CI 0.16‐0.25) [44].

Gender was assessed in 100% (4/4) of the studies analyzing telemedicine and remote primary care [33,36,44,45], with 75% (3/4) reporting higher usage among women [33,44,45]. In Sweden, women accounted for 65.4% of users (IRR 1.39, 95% CI 1.28‐1.51) [44], while one study found no gender difference [36].

Socioeconomic factors were assessed in 100% (4/4) of the studies [33,36,44,45]. Higher education was associated with greater use (OR 1.41, 95% CI 1.19‐1.67) [36], with postsecondary users showing the highest engagement (7.2%; OR 1.51, 95% CI 1.74‐1.56) [33]. More users were observed among individuals with higher incomes (7.7% vs 4.0%; OR 2.46, 95% CI 2.38‐2.54) [33]. Employment was also linked to usage: 85.7% of users were employed versus 14.3% unemployed [45], while one study reported lower odds among retirees (OR 0.68, 95% CI 0.46‐1.02) [36]. One study found no differences across social class [44].

Cultural background and origin were assessed in 50% (2/4) of the studies, both of which were based on large population-based datasets [33,45]. Both studies reported lower uptake among foreign-born users compared to natives (3.2% vs 7.2%; OR 0.53, 95% CI 0.52‐0.55) [33].

Urban residency was analyzed in 2 [33,45] of 4 studies [33,36,44,45]. One study associated it with higher use [45], while residing ≥10 km from health care services was significantly associated with higher telemedicine use (7.1%; OR≤1.11) [33].

Health status was analyzed in 100% (4/4) of the studies [33,36,44,45], showing mixed results. Better health predicted higher use (OR 1.31, 95% CI 1.01‐1.69) [36]. In contrast, heart failure was associated with lower use (OR 0.49, 95% CI 0.40‐0.59) [33]. Some conditions, such as depression, asthma, and chronic obstructive pulmonary disease, were linked to higher use, with greater morbidity also associated with increased engagement (IRR 2.67, 95% CI 2.02‐3.54) [44,45].

### Online Appointment Booking

Inequalities in online appointment booking were examined in 11% (2/18) of the studies [41,48], both showing that younger adults were more likely to use these systems. In Finland, use declined sharply with age [41], while in England, practices with more patients aged 15‐34 years had slightly higher booking rates (+1.35%) [48]. Gender differences were assessed in 100% (2/2) of the studies. Both studies reported lower online booking among men [41,48].

Socioeconomic and cultural factors were analyzed in 50% (1/2) of the studies, revealing lower booking rates in practices located in more deprived areas, as well as reduced uptake among non-White populations [48].

Health status was assessed in 50% (1/2) of the studies, showing that patients with long-term conditions booked less frequently (–0.77%) [48].

Finally, digital competence was evaluated in 50% (1/2) of the studies and was strongly associated with use; patients with good digital skills were over 10 times more likely to book online (OR 10.91, 95% CI 7.24‐16.44) [41].

## Discussion

### Principal Findings

Although European public health care systems are characterized by universal health care coverage, this systematic review aimed to examine inequalities in access to and use of eHealth tools across social determinants of health. The findings reveal persistent and significant social inequalities, with lower usage consistently reported among older adults, individuals with lower socioeconomic or educational status, rural populations, ethnic minorities, people with poor health, and those with limited digital competence. Findings regarding gender were more mixed, with some studies reporting slightly lower usage among men. The majority of included studies were methodologically robust, with most rated high quality and a few moderate quality according to JBI appraisal and conducted with widely varying sample sizes, which should be considered when interpreting these patterns and their potential translation into real-world health care practice. eHealth portals were the most investigated tool, but EHRs and telemedicine showed more consistent evidence of social gradients. Online appointment booking was the least studied category, limiting the strength of conclusions in this area.

### Interpretation of Key Findings

Although digitalization in health care has been presented as improving access and system efficiency [1,2,50], our findings reveal a generational gap across all tool types [32,33,35-39,41,42,44-46,48,49]. Lower usage among older adults reflects not only differences in digital competence—such as less experience and lower technological confidence [51,52]—but also a lack of service adaptation to their needs and potential age-related cognitive barriers that limit effectiveness [53]. These observations, derived mostly from high-quality studies, strengthen the reliability of the age-related patterns observed. They were consistently found both in medium-sized samples [37] and in large administrative datasets [33,44], reinforcing the robustness of these patterns across study designs. At the same time, heterogeneity in measurement and study scale reduces the precision of effect estimates, particularly in smaller studies. In addition, unintuitive interfaces, low perceived usefulness, and a preference for in-person care further contribute to lower uptake [54]. While family support may partially mitigate this gap [54,55], findings are inconsistent [40,56]. This raises concerns about reduced autonomy, informal caregiver burden—often falling on women—and the exclusion of those without social support.

Gender-related patterns were less consistent. Some studies showed higher usage among women for EHRs and telemedicine, while differences in other services were weaker, albeit with a general trend toward greater women’s engagement. These mixed results may reflect contextual factors such as national health care organization, cultural norms, and sample composition. Some null findings came from smaller or moderate samples [32,40], whereas large studies such as Dahlgren et al [33] consistently showed gender differences. Greater women’s involvement may be interpreted in light of traditional gender roles, which assign women the responsibility for caregiving, reflected in a higher willingness to seek care, familiarity with health services, and confidence in sustained, active communication [57] as well as in more continuous use, as suggested by one of the included studies [46]. However, previous literature shows that in certain contexts, the gender gap is reversed, possibly due to broader disparities in digital engagement. Women tend to use a more limited range of digital services and engage with them less frequently and intensively than men, reflecting differences in digital familiarity and confidence [58,59].

It is well established that the socioeconomic gradient not only determines access to material resources but also shapes individuals’ ability to navigate digital environments. In our review, educational attainment, income level, and residence in disadvantaged areas were systematically associated with lower use of digital health tools, in line with studies from other digitalizing systems [60]. Evidence regarding employment status was less consistent, with some studies suggesting lower use among unemployed individuals. Beyond material barriers (access to internet or digital devices), differences in digital literacy, confidence, and prior experience—the so-called third-level digital divide—further perpetuated exclusion [61,62]. Although education strongly predicted competences, structural conditions such as local digital infrastructure and social position also shaped digital engagement. In disadvantaged regions, limited digital services may affect both patients and health care professionals, potentially reinforcing existing health care disparities [63]. Given the overall rigor of the included studies, these findings appear reliable; however, their transferability to different health care contexts should be approached with caution. The WHO stresses that closing this gap requires not only investment in infrastructure, but also training and support strategies specifically targeted at the most vulnerable groups [64]. The consistency of socioeconomic gradients across both small samples [34] and very large datasets [33] strengthens confidence in these associations and suggests that they are not driven by sample-specific variability.

Beyond socioeconomic status, ethnic minorities have shown lower engagement with all the tools analyzed, even after adjusting for socioeconomic variables. Language barriers, lack of familiarity with the health care system, distrust of institutional platforms, and poor cultural adaptation of services emerge as recurring obstacles [46,65,66].

Although urban environments are generally associated with higher use of eHealth portals [39,49], one study showed greater use of video consultations in rural areas [33], consistent with previous literature [67], which suggests that digitalization compensates for limited in-person services. This pattern, particularly in studies with careful design and with large samples such as Dahlgren et al [33], suggests that geographical effects are not artifacts of small-sample instability. However, rural areas often have lower educational attainment, which can reduce digital competence [42,45]. These findings indicate that geography can both mitigate and amplify inequalities, depending on the system’s capacity to adapt.

Regarding health status, some studies showed lower use among people with multimorbidity, in line with previous literature [68,69], reflecting the Inverse Care Law, where populations with greater health care needs often face reduced access [70,71]. However, other studies found higher use among people with poorer health, likely due to more frequent contact with the health care system [40,48]. These mixed results are partly attributable to methodological differences and varying study quality, with patterns more clearly observed in studies with comprehensive data and stronger design [33,37]. Variation in sample sizes also contributed: larger datasets such as Eriksson et al [44] and Dahlgren et al (N≈2 million) [33] produced more stable health-related estimates than smaller clinical samples, helping explain discrepancies in the direction and magnitude of associations. Overall, individuals with greater clinical needs may face additional barriers when poor health intersects with low education or socioeconomic status.

Although the overall methodological quality of the included studies was acceptable—with most rated high quality and a few moderate quality according to the JBI appraisal—the predominance of cross-sectional designs limits causal inference. Substantial heterogeneity in study populations, digital tools, and outcome indicators also affects comparability across studies. Furthermore, the wide variability in sample sizes introduces important differences in estimate precision, which should be taken into account when interpreting inconsistent findings across determinants. These methodological and contextual differences influence how findings can be translated into real-world practice: variations in health care organization, digital infrastructure, and population characteristics across countries may alter the magnitude and direction of observed inequalities. Therefore, interpretation and policy translation should be undertaken with caution, recognizing that local context strongly shapes the impact of the digital divide.

### Limitations and Strengths

This systematic review has several limitations. One limitation is that the search strategy was not developed in collaboration with an information scientist, which might have influenced the sensitivity and comprehensiveness of the retrieved records. The conclusions may also be affected by differences in study design, sample representativeness, and reporting. Although most studies were rated as high quality according to the JBI appraisal, some moderate-quality designs introduce a potential risk of bias that should be considered when interpreting the results. The exclusion of qualitative studies—although methodologically justified to ensure comparability—may have constrained the review’s ability to capture rich contextual insights, such as user experiences, perceived barriers, and cultural or behavioral factors influencing digital health use, which are crucial for understanding the mechanisms behind observed inequalities. Heterogeneity in populations, definitions of eHealth use, and measurement of social determinants limited comparability and prevented meta-analysis, requiring a narrative synthesis. Restricting the search to peer-reviewed publications in English or Spanish may have introduced language and publication bias, excluding relevant evidence published in other languages or in gray literature. Nevertheless, this criterion enhances replicability and scientific robustness. The focus on public health care systems in Europe restricts generalizability to other regions. Additionally, the studies’ highly variable sample sizes, while not undermining the findings, do influence precision and should be considered when weighing the strength of evidence across determinants.

Despite these limitations, the review has several strengths. It followed PRISMA guidelines, used a comprehensive search across 4 major databases, supplemented by backward citation searching, and included independent screening and data extraction by multiple reviewers to improve validity. By synthesizing evidence across diverse European settings, this review offers an up-to-date and policy-relevant overview of eHealth-related inequalities, and its classification by type of digital service enables a more granular understanding of patterns of exclusion. No amendments were made to the registered protocol after PROSPERO registration.

These findings carry important implications for real-world health care policy and practice. By highlighting persistent inequalities in access and use of digital health tools across multiple social determinants, the review underscores the need for targeted interventions, including digital literacy programs, inclusive eHealth platform design, and support strategies for older adults, ethnic minorities, and socioeconomically disadvantaged populations. The innovative contribution of this review lies in its focus on quantitative evidence from European public health care systems, integrating results across diverse social determinants and types of eHealth tools. This provides a policy-relevant, evidence-based perspective to guide equitable digital transformation and foster inclusive health systems.

### Future Research Directions

Future research should explore the long-term effects of digital exclusion on health outcomes, assess the equity impact of digital health policies, and promote participatory research with underserved communities to co-develop inclusive digital solutions. There is a clear need for intersectional frameworks to understand how intersecting inequalities shape digital health access, especially in highly vulnerable populations. Additionally, research is scarce in low-income countries, where sociodemographic and economic contexts may lead to different and sometimes contrasting patterns, for example regarding gender. Further studies are also needed to evaluate the actual impact of digital health tools on access to health care services more broadly—not only their uptake or use but also how they affect entry into the health system and care continuity. Moreover, more attention should be paid to the underlying mechanisms driving these inequalities, to move beyond description and identify structural, institutional, or behavioral factors that generate unequal access. Finally, while the number of studies exploring social disparities in digital health access has increased, there remains a gap in longitudinal and in-depth research that can capture the complexity and dynamics of these inequities over time.

### Conclusions

This systematic review shows that digital transformation in European public health systems is not equally benefiting all population groups. Across the studies analyzed, persistent social inequalities in the use of digital health tools—particularly EHRs, eHealth portals, and telemedicine and remote primary care—were observed. The most excluded from digital care services are older adults, individuals with lower socioeconomic or educational status, ethnic minorities, rural populations, people in poor health, and those with limited digital competence.

Rather than closing gaps, digitalization may replicate or deepen existing health inequalities if equity is not explicitly addressed. Ensuring inclusive design, supporting digital literacy, and engaging underserved communities are essential steps to make digital health a tool for reducing—not reinforcing—structural exclusion. Although a few studies explicitly explored interactions between determinants, the detected patterns suggest that the combination of factors such as advanced age, low socioeconomic status, migrant background, and limited digital competence tends to produce cumulative effects that significantly limit the adoption of eHealth tools. In interpreting these findings, both the heterogeneity of studies and their risk of bias should be taken into account. While these findings offer a policy-relevant perspective, the heterogeneity of study designs, populations, and outcomes, as well as the risk of bias, limits direct translation into policy and practice. By synthesizing quantitative evidence across diverse social determinants and types of digital health tools, this review not only highlights persistent inequalities but also offers a policy-relevant perspective. These findings can inform equity-oriented interventions, guide inclusive eHealth design, and support digital literacy programs, helping policymakers and health systems ensure that digital transformation benefits all population groups.

## Supplementary material

10.2196/81841 Multimedia Appendix 1 Complete search strategies.

10.2196/81841 Multimedia Appendix 2 Study-specific definitions of determinants and eHealth use.

10.2196/81841 Multimedia Appendix 3 Risk of bias assessment of each study.

10.2196/81841 Multimedia Appendix 4 Extended evidence table summarizing the main information of each study included in the systematic review.

10.2196/81841 Checklist 1 PRISMA checklist.

## Abbreviations

EHR: electronic health record
JBI: Joanna Briggs Institute
OR: odds ratio
PRISMA: Preferred Reporting Items for Systematic Reviews and Meta-Analyses
PRISMA-S: Preferred Reporting Items for Systematic Reviews and Meta-Analyses literature search extension
WHO: World Health Organization
