# Supplementary material for: Digital Inequalities in the Use of eHealth Services in European Public Health Care Systems: Systematic Review of Observational Studies
Source: J Med Internet Res. 2026 Feb 9;28:e81841. doi: 10.2196/81841 (PMC12885193; doi:10.2196/81841)
Supplement: Multimedia Appendix 1 [file jmir-v28-e81841-s001.docx]

**Multimedia Appendix 1.** Complete search strategies.

**PUBMED: 10 year and Spanish or English filter**

((("telemedicine"[MeSH Terms] OR "telemedicine"[Title/Abstract] OR "telehealth"[Title/Abstract] OR "eHealth"[Title/Abstract] OR "mHealth"[Title/Abstract] OR "Digital Health" [MeSH Terms] OR "digital health*"[Title/Abstract] OR "digital primary care"[Title/Abstract] OR "digital technolog*"[Title/Abstract] OR "ePrescription"[Title/Abstract] OR "telecare"[Title/Abstract] OR "patient portal*"[Title/Abstract] OR "mobile health"[Title/Abstract] OR "teleconsult*"[Title/Abstract] OR "online consult*"[Title/Abstract] OR "consultation request"[Title/Abstract] OR "eVisit"[Title/Abstract] OR "tele referral*"[Title/Abstract] OR "e-referrals"[Title/Abstract] OR "remote consultation*"[Title/Abstract] OR "digital consultation*"[Title/Abstract] OR "digitalization"[Title/Abstract])
**AND** ("health inequities"[Title/Abstract] OR "health disparities"[Title/Abstract] OR "healthcare disparities"[Title/Abstract] OR "digital divide"[Title/Abstract] OR "inequalit*"[Title/Abstract] OR "equit*"[Title/Abstract] OR "inequit*"[Title/Abstract] OR "disparit*"[Title/Abstract] OR "differences"[Title/Abstract] OR "gap"[Title/Abstract])
**AND** ("factors associated"[Title/Abstract] OR "determinants"[Title/Abstract] OR "user profile"[Title/Abstract] OR "user type"[Title/Abstract] OR "socioeconomic"[Title/Abstract] OR "sociodemographic"[Title/Abstract] OR "socio-demographic"[Title/Abstract] OR "income"[Title/Abstract] OR "educational status"[Title/Abstract] OR "deprivation"[Title/Abstract] OR "age"[Title/Abstract] OR "aged"[Title/Abstract] OR "elderly"[Title/Abstract] OR "older"[Title/Abstract] OR "gender"[Title/Abstract] OR "sex"[Title/Abstract] OR "rural"[Title/Abstract] OR "migrant"[Title/Abstract] OR "foreign"[Title/Abstract] OR "minorities"[Title/Abstract] OR "ethnic*"[Title/Abstract] OR "disabled"[Title/Abstract] OR "disabilit*"[Title/Abstract])
**AND** ("Delivery of Health Care"[MeSH Major Topic] OR "eHealth access"[Title/Abstract] OR "access*"[Title/Abstract] OR "accessibility"[Title/Abstract] OR "utilization"[Title/Abstract] OR "use"[Title/Abstract] OR "user*"[Title/Abstract] OR "non-user"[Title/Abstract])
**AND** ("europe*"[Title/Abstract] OR "austria"[Title/Abstract] OR "belgium"[Title/Abstract] OR "croatia"[Title/Abstract] OR "cyprus"[Title/Abstract] OR "czechia"[Title/Abstract] OR "denmark"[Title/Abstract] OR "estonia"[Title/Abstract] OR "finland"[Title/Abstract] OR "france"[Title/Abstract] OR "germany"[Title/Abstract] OR "greece"[Title/Abstract] OR "hungary"[Title/Abstract] OR "ireland"[Title/Abstract] OR "italy"[Title/Abstract] OR "latvia"[Title/Abstract] OR "lithuania"[Title/Abstract] OR "luxembourg"[Title/Abstract] OR "malta"[Title/Abstract] OR "netherlands"[Title/Abstract] OR "poland"[Title/Abstract] OR "portugal"[Title/Abstract] OR "romania"[Title/Abstract] OR "slovakia"[Title/Abstract] OR "slovenia"[Title/Abstract] OR "spain"[Title/Abstract] OR "sweden"[Title/Abstract] OR "united kingdom"[Title/Abstract] OR "UK"[Title/Abstract] OR "great britain"[Title/Abstract] OR "england"[Title/Abstract] OR "wales"[Title/Abstract] OR "scotland"[Title/Abstract] OR "northern ireland"[Title/Abstract] OR "iceland"[Title/Abstract] OR "norway"[Title/Abstract] OR "liechtenstein"[Title/Abstract] OR "switzerland"[Title/Abstract] OR "european union"[Title/Abstract]))

**NOT** ("COVID-19"[Title/Abstract] OR "covid19"[Title/Abstract] OR "qualitative study"[Title/Abstract] OR "qualitative research"[Title/Abstract] OR "clinical trial"[Publication Type] OR "review"[Publication Type] OR "systematic review"[Publication Type]))

**SCOPUS: 10 year and Spanish or English filter**

**TITLE-ABS-KEY** ("telemedicine" OR "telehealth" OR "eHealth" OR "mHealth" OR "digital health*" OR "digital primary care" OR "digital technolog*" OR "ePrescription" OR "telecare" OR "patient portal*" OR "mobile health" OR "teleconsult*" OR "online consult*" OR "consultation request" OR "eVisit" OR "tele-referral*" OR "e-referrals" OR "remote consultation*" OR "digital consultation*" OR "digitalization" OR "video consult*")

**AND** **TITLE-ABS-KEY** ("health disparities" OR "healthcare disparities" OR "digital divide" OR "inequalit*" OR "equit*" OR "inequit*" OR "disparit*" OR "differences" OR "gap")

**AND** **TITLE-ABS-KEY** ( "factors associated" OR "determinants" OR "user profile" OR "user type" OR "socioeconomic" OR "sociodemographic" OR "socio-demographic" OR "income" OR "educational status" OR "deprivation" OR "gender" OR "sex" OR "rural" OR "age" OR "aged" OR "elderly" OR "older" OR "migrant" OR "foreign" OR "minorities" OR "ethnic*" OR "disabled" OR "disabilit*")

**AND TITLE-ABS-KEY** ( "delivery of health care" OR "access*" OR "accessibility" OR "utilization" OR "use" OR "user*" OR "non-user")

**AND TITLE-ABS-KEY** ( "Europe*" OR "austria" OR "belgium" OR "croatia" OR "cyprus" OR "czechia" OR "denmark" OR "estonia" OR "finland" OR "france" OR "germany" OR "greece" OR "hungary" OR "ireland" OR "italy" OR "latvia" OR "lithuania" OR "luxembourg" OR "malta" OR "netherlands" OR "poland" OR "portugal" OR "romania" OR "slovakia" OR "slovenia" OR "spain" OR "sweden" OR "united kingdom" OR "UK" OR "great britain" OR "england" OR "wales" OR "scotland" OR "northern ireland" OR "iceland" OR "norway" OR "liechtenstein" OR "switzerland" OR "european union")

**AND NOT TITLE-ABS-KEY (** "covid 19" OR "COVID-19")

**AND NOT TITLE-ABS-KEY** ( "qualitative study" OR "qualitative research" OR "review" OR "systematic review" OR "clinical trial")

**WOS: 10 year and Spanish or English filter**

**TS**=("telemedicine" OR "telehealth" OR "eHealth" OR "mHealth" OR "digital health*" OR "digital primary care" OR "digital technolog*" OR "ePrescription" OR "telecare" OR "patient portal*" OR "mobile health" OR "teleconsult*" OR "online consult*" OR "consultation request" OR "eVisit" OR "tele-referral*" OR "e-referrals" OR "remote consultation*" OR "digital consultation*" OR "digitalization")

**AND TS**=("health disparities" OR "healthcare disparities" OR "digital divide" OR "inequalit*" OR "equit*" OR "inequit*" OR "disparit*" OR "differences" OR "gap")

**AND TS**=("factors associated" OR "determinants" OR "user profile" OR "user type" OR "socioeconomic" OR "sociodemographic" OR "socio-demographic" OR "income" OR "educational status" OR "deprivation" OR "gender" OR "sex" OR "older" OR "age" OR "aged" OR "elderly" OR "rural" OR "migrant" OR "foreign" OR "minorities" OR "ethnic*" OR "disabled" OR "disabilit*")

**AND TS**=("delivery of health care" OR "access*" OR "accessibility" OR "utilization" OR "use" OR "user*" OR "non-user")

**AND TS**=("europe*" OR "austria" OR "belgium" OR "croatia" OR "cyprus" OR "czechia" OR "denmark" OR "estonia" OR "finland" OR "france" OR "germany" OR "greece" OR "hungary" OR "ireland" OR "italy" OR "latvia" OR "lithuania" OR "luxembourg" OR "malta" OR "netherlands" OR "poland" OR "portugal" OR "romania" OR "slovakia" OR "slovenia" OR "spain" OR "sweden" OR "united kingdom" OR "UK" OR "great britain" OR "england" OR "wales" OR "scotland" OR "northern ireland" OR "iceland" OR "norway" OR "liechtenstein" OR "switzerland" OR "european union")

**NOT TS**=("covid 19" OR "COVID-19")

**NOT TS**=("qualitative study" OR "qualitative research" OR "review" OR "systematic review" OR "clinical trial")

**PsycINFO (ProQuest): 10 year and Spanish or English filter**

telemedicine OR telehealth OR eHealth OR mHealth OR "digital health*" OR "digital primary care" OR "digital technolog*" OR ePrescription OR telecare OR "patient portal*" OR "mobile health" OR teleconsult* OR "online consult*" OR "consultation request" OR eVisit OR "tele referral*" OR "e-referrals" OR "remote consultation*" OR "digital consultation*" OR digitalization **NOFT**

"health inequities" OR "health disparities" OR "healthcare disparities" OR "digital divide" OR inequalit* OR equit* OR inequit* OR disparit* OR differences OR gap **NOFT**

"factors associated" OR determinants OR "user profile" OR "user type" OR socioeconomic OR sociodemographic OR "socio-demographic" OR income OR "educational status" OR deprivation OR age OR aged OR elderly OR older OR gender OR sex OR rural OR migrant OR foreign OR minorities OR ethnic* OR disabled OR disabilit* **NOFT**

"eHealth access" OR access* OR accessibility OR utilization OR use OR user* OR "non-user" **NOFT**

europe* OR austria OR belgium OR croatia OR cyprus OR czechia OR denmark OR estonia OR finland OR france OR germany OR greece OR hungary OR ireland OR italy OR latvia OR lithuania OR luxembourg OR malta OR netherlands OR poland OR portugal OR romania OR slovakia OR slovenia OR spain OR sweden OR "united kingdom" OR UK OR "great britain" OR england OR wales OR scotland OR "northern ireland" OR iceland OR norway OR liechtenstein OR switzerland OR "european union" **NOFT**

**NOT**

COVID-19 OR "covid 19" OR "qualitative study" OR "qualitative research" OR "clinical trial" OR review OR "systematic review" **NO**
