# Supplementary material for: Digital Inequalities in the Use of eHealth Services in European Public Health Care Systems: Systematic Review of Observational Studies
Source: J Med Internet Res. 2026 Feb 9;28:e81841. doi: 10.2196/81841 (PMC12885193; doi:10.2196/81841)
Supplement: Multimedia Appendix 3 [file jmir-v28-e81841-s003.docx]

**Multimedia Appendix 3.** Ris of Bias assessment of each study.

| **Author/year** | **Design** | **Q^a^1** | **Q2** | **Q3** | **Q4** | **Q5** | **Q6** | **Q7** | **Q8** | **Q9** | **Q10** | **Q11** | **Nº YES** | **Quality** |
| --- | --- | --- | --- | --- | --- | --- | --- | --- | --- | --- | --- | --- | --- | --- |
| **CROSS SECTIONAL STUDIES** | | | | | | | | | | | | | | |
| **González‑Cacheda et al. (2025)** | Cross Sectional | No | Yes | Unclear | No | Yes | Yes | Unclear | Yes |  |  |  | 4 | Moderate |
| **Hörhammer et al. (2025)** | Cross-sectional | Yes | Yes | Yes | Yes | Yes | Unclear | Yes | Yes |  |  |  | 7 | High |
| **Knöchelmann et al. (2024)** | Cross Sectional | Yes | Yes | Unclear | Unclear | Yes | Yes | Unclear | Yes |  |  |  | 5 | Moderate |
| **Söderberg et al.(2024)** | Cross-sectional | Yes | Yes | Yes | Yes | Yes | Yes | Yes | Yes |  |  |  | 8 | High |
| **Wilkens et al. (2024)** | Cross-sectional | Yes | Yes | Yes | Yes | Yes | Yes | Yes | Yes |  |  |  | 8 | High |
| **Muli et al. (2024)** | Cross-sectional | Yes | Yes | Yes | Yes | Yes | Yes | Yes | Yes |  |  |  | 8 | High |
| **Zhang et al. (2023)** | Cross-sectional | Yes | No | Yes | Yes | Yes | Yes | Yes | Yes |  |  |  | 7 | High |
| **Pierce et al. (2023)** | Cross-sectional | Yes | Unclear | Yes | Yes | Yes | Yes | Yes | Yes |  |  |  | 7 | High |
| **Heponiemi et al. (2022)** | Cross Sectional | Yes | Yes | Yes | Yes | Yes | Yes | Yes | Yes |  |  |  | 8 | High |
| **Chapman et al. (2022)** | Cross Sectional | Yes | Yes | Yes | Yes | Yes | Yes | Yes | Yes |  |  |  | 8 | High |
| **Neves et al. (2021)** | Cross Sectional | Yes | Yes | Yes | Yes | Yes | Yes | Yes | Yes |  |  |  | 8 | High |
| **Dahlgren et al. (2021)** | Cross Sectional | Yes | Yes | Yes | Yes | Yes | Yes | Yes | Yes |  |  |  | 8 | High |
| **Merkel et al. (2020)** | Cross Sectional | Yes | Yes | Yes | Yes | Yes | Yes | Yes | Yes |  |  |  | 8 | High |
| **PREVALENCE STUDIES** | | | | | | | | | | | | | | |
| **Kharko et al. (2025)** | Prevalence study | Yes | Yes | Yes | Yes | Yes | No | Yes | Yes | No |  |  | 7 | High |
| **Pálsdóttir et al. (2024)** | Prevalence study | Yes | Yes | Yes | Yes | Yes | Yes | Yes | Yes | Yes |  |  | 9 | High |
| **Petersen et al. (2017)** | Prevalence Study | Yes | Yes | Yes | Yes | Unclear | Yes | Yes | No | Unclear |  |  | 6 | Moderate |
| **COHORT STUDIES** | | | | | | | | | | | | | | |
| **Eriksson et al. (2025)** | Cohort study | Yes | Yes | Yes | Yes | Yes | Yes | Yes | Yes | Yes | Yes |  | 10 | High |
| **ECOLOGICAL STUDIES** | | | | | | | | | | | | | | |
| **Kc et al. (2024)** | Ecological (cohort) | Yes | Yes | Yes | No | No | Yes | Yes | Yes | Unclear | No | Yes | 5 | Moderate |

Note: Studies were assessed using the corresponding JBI critical appraisal checklist for each study design (cross-sectional, prevalence, or cohort). Studies are grouped by design to enhance readability and facilitate comparison of quality ratings. ^a^ Q: each Q corresponds to the number of each question in the corresponding JBI tool for each study.
