# Supplementary material for: Digital Inequalities in the Use of eHealth Services in European Public Health Care Systems: Systematic Review of Observational Studies
Source: J Med Internet Res. 2026 Feb 9;28:e81841. doi: 10.2196/81841 (PMC12885193; doi:10.2196/81841)
Supplement: Multimedia Appendix 4 [file jmir-v28-e81841-s004.docx]

**Multimedia Appendix 4.** Extended evidence table summarizing the main information of each study included in the systematic review.

| **Author/ country/ year** | **Study design (1), population (2), and statistical analysis (3) and eHeealth tools (4)** | **Outcome** | **Social Determinants of Health Assessed** | | | | **Numeric Main Findings on Inequalities** | | | | | | | | | | | | | | | | | | | | | | | **Description of main findings in the study’s main text** |
| --- | --- | --- | --- | --- | --- | --- | --- | --- | --- | --- | --- | --- | --- | --- | --- | --- | --- | --- | --- | --- | --- | --- | --- | --- | --- | --- | --- | --- | --- | --- |
| González‑Cacheda et al. (2025) Spain  [49] | 1. Cross-sectional  2. Population present in the  Health Barometer 2022 (N = 7,454)  3. Univariate Logistic Regressions  4. EHRs^c^ | Use of medical records via internet |  | | | | Medical records (use) | | | | | | | | | | | | | | | | | | | | | | | People with a higher level of digital experience in the everyday environment were more likely to use the digital medical record. Effective use of the digital medical record appeared to be among young and middle-aged people. People of Spanish nationality were more likely to use digital medical records. |
|  |  |  |  |  |  |  | Yes (%) | | | | | | | No (%) | | | | | | | | Logistic regression for use OR^b^ | | | | | | | |  |
|  |  |  | Gender | | 7358 | |  | | | | | | |  | | | | | | | | 1.05 | | | | | | | |  |
|  |  |  |  |  | Men | | 29.1 | | | | | | | 70.9 | | | | | | | |  |  |  |  |  |  |  |  |  |
|  |  |  |  |  | Woman | | 28.6 | | | | | | | 71.4 | | | | | | | |  |  |  |  |  |  |  |  |  |
|  |  |  | Age | | 7358 | |  | | | | | | |  | | | | | | | | 0.86^a^ | | | | | | | |  |
|  |  |  |  |  | 18–24 | | 32 | | | | | | | 68 | | | | | | | |  |  |  |  |  |  |  |  |  |
|  |  |  |  |  | 25–34 | | 41.6 | | | | | | | 58.4 | | | | | | | |  |  |  |  |  |  |  |  |  |
|  |  |  |  |  | 35–44 | | 35.3 | | | | | | | 64.7 | | | | | | | |  |  |  |  |  |  |  |  |  |
|  |  |  |  |  | 45–54 | | 33.9 | | | | | | | 66.1 | | | | | | | |  |  |  |  |  |  |  |  |  |
|  |  |  |  |  | 55–64 | | 26.8 | | | | | | | 73.2 | | | | | | | |  |  |  |  |  |  |  |  |  |
|  |  |  |  |  | 65–74 | | 19.3 | | | | | | | 80.7 | | | | | | | |  |  |  |  |  |  |  |  |  |
|  |  |  |  |  | Over 75 | | 6.1 | | | | | | | 93.9 | | | | | | | |  |  |  |  |  |  |  |  |  |
|  |  |  | Education | | 7293 | |  | | | | | | |  | | | | | | | | 1.41 | | | | | | | |  |
|  |  |  |  |  | Without studies | | 1 | | | | | | | 99 | | | | | | | |  |  |  |  |  |  |  |  |  |
|  |  |  |  |  | Primary | | 8.5 | | | | | | | 91.5 | | | | | | | |  |  |  |  |  |  |  |  |  |
|  |  |  |  |  | Secondary education (1st stage) | | 16.2 | | | | | | | 83.8 | | | | | | | |  |  |  |  |  |  |  |  |  |
|  |  |  |  |  | Secondary education (2nd stage) | | 27.7 | | | | | | | 72.3 | | | | | | | |  |  |  |  |  |  |  |  |  |
|  |  |  |  |  | Vocational training | | 31.7 | | | | | | | 68.3 | | | | | | | |  |  |  |  |  |  |  |  |  |
|  |  |  |  |  | University studies | | 40.4 | | | | | | | 59.6 | | | | | | | |  |  |  |  |  |  |  |  |  |
|  |  |  | Nationality | | 7358 | |  | | | | | | |  | | | | | | | | 1.45^a^ | | | | | | | |  |
|  |  |  |  |  | Spain National | | 29.1 | | | | | | | 70.9 | | | | | | | |  |  |  |  |  |  |  |  |  |
|  |  |  |  |  | Foreign | | 23.3 | | | | | | | 76.7 | | | | | | | |  |  |  |  |  |  |  |  |  |
|  |  |  | Class | | 6394 | |  | | | | | | |  | | | | | | | | 0.94^a^ | | | | | | | |  |
|  |  |  |  |  | Upper/ upper- middle | | 41 | | | | | | | 59 | | | | | | | |  |  |  |  |  |  |  |  |  |
|  |  |  |  |  | Middle | | 32.3 | | | | | | | 67.7 | | | | | | | |  |  |  |  |  |  |  |  |  |
|  |  |  |  |  | Medium–low | | 27.6 | | | | | | | 72.4 | | | | | | | |  |  |  |  |  |  |  |  |  |
|  |  |  |  |  | Working/ proletariat | | 28.9 | | | | | | | 71.1 | | | | | | | |  |  |  |  |  |  |  |  |  |
|  |  |  |  |  | Low/poor | | 19.6 | | | | | | | 80.4 | | | | | | | |  |  |  |  |  |  |  |  |  |
|  |  |  | Device use | | 7347 | |  | | | | | | |  | | | | | | | | 1.51^a^ | | | | | | | |  |
|  |  |  |  |  | Yes | | 37.4 | | | | | | | 62.6 | | | | | | | |  |  |  |  |  |  |  |  |  |
|  |  |  |  |  | No | | 23.3 | | | | | | | 76.7 | | | | | | | |  |  |  |  |  |  |  |  |  |
| Kharko et al. (2025) Norway, Sweden, Finland  [48] | 1. Prevalence study  2. NORDeHEALTH 2022  Survey  (N = 27,038)  3. Descriptive  4. EHRs | Access to EHRs |  | | | | Accessed EHR in the last 12 months, n (%) | | | | | | | | | | | | | | | | | | | | | | | Women were significantly less likely than men to had never accessed the EHRs or be first-timers, and were more likely to have visited over 20 times, χ2 = 57, df = 4,*P* < .001. |
|  |  |  |  |  |  |  | Never accessed or first time | | | | | | 2-9 times | | | | | 10-20 times | | | | | | More than 20 times | | | | | |  |
|  |  |  | Sex | | Women | | 798 (4.3) | | | | | | 7163 (39) | | | | | 4661 (25.4) | | | | | | 5759 (31.3) | | | | | |  |
|  |  |  |  | | Men | | 539 (6.2) | | | | | | 3369 (39) | | | | | 2242 (26) | | | | | | 2488 (28.8) | | | | | |  |
| Eriksson et al. (2025) Sweden  [35] | 1. Retrospective cohort (registry-based)  2. Individuals of any age registered with Primary Health Care Centres, and having made at least 1 outpatient consultation during the study period (N = 73,486)  3. Multivariable Logistic Regression  4. Telemedicine and Remote Primary Care | Use of telemedicine consultation in primary healthcare and EHRs |  | | | | User  n=2,500  n(%) | | | | | | | Non-users  n=70,899  n(%) | | | | | | | | Logistic regression for use  IRR^d^ (CI 95%) | | | | | | | | Women were more likely to be users of telemedicine compared to men (*P* <.001). Furthermore, the results suggest that patients belonging to age group 20–39 were more likely to be users of telemedicine services compared to the reference group (*P* <.001). Age groups 60–79 and 80+ were less likely to be users of telemedicine compared to the reference group (*P* <.001). Patients belonging to Resource utilization Band groups 2–5 were more likely to be users of telemedicine compared to the reference (*P* < .001). The mean Resurce Utilization Band value in the two populations was computed to 2.1 among users and 1.9 among non-users (*P* <.001). Furthermore, the results suggest a positive relationship between the number of EHRs entries and the likelihood of being a user of telemedicine, with an IRR of 1.5 (*P* <.001). Regarding Care Need Index, the t-test did not indicate any statistically significant differences between users and non-users of telemedicine. |
|  |  |  | Sex | | Men | | 993 (40) | | | | | | | 36,541 (51) | | | | | | | | Ref | | | | | | | |  |
|  |  |  |  |  | Women | | 1507 (60) | | | | | | | 34,445 (49) | | | | | | | | 1.39 (1.28-1.51) ^a^ | | | | | | | |  |
|  |  |  | Age | | 0–19 | | 479 (19) | | | | | | | 14,984 (21) | | | | | | | | Ref | | | | | | | |  |
|  |  |  |  |  | 20–39 | | 978 (39) | | | | | | | 16,760 (24) | | | | | | | | 1.64 (1.51-1.78) ^a^ | | | | | | | |  |
|  |  |  |  |  | 40–59 | | 645 (26) | | | | | | | 16,846 (24) | | | | | | | | 1 (0.92-1.11) | | | | | | | |  |
|  |  |  |  |  | 60–79 | | 346 (14) | | | | | | | 17,359 (24) | | | | | | | | 0.45 (0.40-0.50) ^a^ | | | | | | | |  |
|  |  |  |  |  | 80+ | | 52 (2) | | | | | | | 5037 (7) | | | | | | | | 0.19 (0.16-0.25) ^a^ | | | | | | | |  |
|  |  |  | Resource Utilization Band | | 0 | | 462 (18) | | | | | | | 15,803 (22) | | | | | | | | Ref | | | | | | | |  |
|  |  |  |  |  | 1 | | 237 (9) | | | | | | | 8098 (11) | | | | | | | | 0.93 (0.82-1.04) | | | | | | | |  |
|  |  |  |  |  | 2 | | 617 (25) | | | | | | | 16,902 (24) | | | | | | | | 1.23 (1.13-1.35) ^a^ | | | | | | | |  |
|  |  |  |  |  | 3 | | 995 (40) | | | | | | | 25,030 (35) | | | | | | | | 1.76 (1.62-1.91) ^a^ | | | | | | | |  |
|  |  |  |  |  | 4 | | 139 (6) | | | | | | | 4075 (6) | | | | | | | | 2.09 (1.81-2.42) ^a^ | | | | | | | |  |
|  |  |  |  |  | 5 | | 28 (1) | | | | | | | 991 (1) | | | | | | | | 2.67 (2.02-3.54) ^a^ | | | | | | | |  |
|  |  |  |  |  | Missing | | 22 (1) | | | | | | | 87 (<1) | | | | | | | | - | | | | | | | |  |
|  |  |  | Care Need Index by sex and age group | | | |  | | | | | | |  | | | | | | | | - | | | | | | | |  |
|  |  |  | Women | | 0–19 | | 0.812 | | | | | | | 0.693 | | | | | | | |  |  |  |  |  |  |  |  |  |
|  |  |  | Women | | 20–39 | | 1.289 | | | | | | | 1.227 | | | | | | | |  |  |  |  |  |  |  |  |  |
|  |  |  | Women | | 40–59 | | 1.063 | | | | | | | 0.97 | | | | | | | |  |  |  |  |  |  |  |  |  |
|  |  |  | Women | | 60–79 | | 0.936 | | | | | | | 1.029 | | | | | | | |  |  |  |  |  |  |  |  |  |
|  |  |  | Women | | 80+ | | 2.074 | | | | | | | 1.925 | | | | | | | |  |  |  |  |  |  |  |  |  |
|  |  |  | Men | | 0–19 | | 0.859 | | | | | | | 0.7 | | | | | | | |  |  |  |  |  |  |  |  |  |
|  |  |  | Men | | 20–39 | | 1.135 | | | | | | | 1.192 | | | | | | | |  |  |  |  |  |  |  |  |  |
|  |  |  | Men | | 40–59 | | 0.807 | | | | | | | 0.938 | | | | | | | |  |  |  |  |  |  |  |  |  |
|  |  |  | Men | | 60–79 | | 0.836 | | | | | | | 0.273 | | | | | | | |  |  |  |  |  |  |  |  |  |
|  |  |  | Men | | 80+ | | 1.651 | | | | | | | 1.074 | | | | | | | |  |  |  |  |  |  |  |  |  |
|  |  |  | Out-patient contacts | | Total number of EHR entries | | 1,13,305 | | | | | | | 23,53,606 | | | | | | | | 1.5 (1.32-1.72) ^a^ | | | | | | | |  |
|  |  |  |  |  | EHR entries per patient | | 45 | | | | | | | 33 | | | | | | | | - | | | | | | | |  |
| Hörhammer et al. (2025) Finland  [36] | 1. Cross-sectional2. Patients from a Finnish mental health and substance abuse unit (N = 438)  3. Univariate and Multivariate Logistic Regressions  4. eHealth portal | Use of digital services (appointment booking, symptom scales, video visit, record access, medical history form, information and self-help programs, therapies, and group appointments) |  | | | | Logistic regression for use | | | | | | | | | | | | | | | | | | | | | | | Higher levels of health confidence and patients aged 65 years or older were likely not to use a digital service. |
|  |  |  |  |  |  |  | Univariate analysis  OR (CI 95%) | | | | | | | | | | | Multivariate analysis  OR (CI 95%)) | | | | | | | | | | | |  |
|  |  |  | Health confidence | | | | 0.61 (0.4-0.84) | | | | | | | | | | | 0.62 (0.45-0.86) ^a^ | | | | | | | | | | | |  |
|  |  |  | Employment status | | Non-employed or unable to work | | 1.01 (0.64-1.59) | | | | | | | | | | | 0.76 (0.45-1.27) | | | | | | | | | | | |  |
|  |  |  | Age | | Age 65 or older | | 0.55 (0.32-0.95) | | | | | | | | | | | 0.40 (0.21-0.75) ^a^ | | | | | | | | | | | |  |
|  |  |  | Sex | | Man gender | | 1.16 (0.71-1.87) | | | | | | | | | | | 0.97 (0.58-1.62) | | | | | | | | | | | |  |
|  |  |  | Duration of care | | | | 1.10 (0.89-1.36) | | | | | | | | | | | 1.10 (0.881.38) | | | | | | | | | | | |  |
| Mul et al. (2024) Sweden  [34] | 1. Cross-sectional  2. Patients ≥18 and guardians of minors living in Region Stockholm who had a consultation with a physician  (N = 3,421)  3. Multivariate Logistic Regressions  4. EHRs | Access to patient-accessible electronic health records |  | | | | Has never read  n(%)  632 (23) | | | | | | | Has read  n(%)  2086 (77) | | | | | | | | Logistic regression for reading  OR (CI 95%) | | | | | | | | The 77% (n=2086) of the participants had read their records (readers), while 23% (n=632) had not read them (non-readers). These findings indicate a high level of awareness among the population studied. The comparison of readers and non-readers showed a larger proportion of females among readers than among non-readers. Readers of Patient-accesible electronic health records were on average younger than non-readers with a mean age of 48 years and had a larger proportion of participants with university or college education and partnered participants compared to non-readers. |
|  |  |  | Gender ^a^ | | Women | | 1473 (71) | | | | | | | 399 (64) | | | | | | | | - | | | | | | | |  |
|  |  |  | Age ^a^ | | Mean | | 48 | | | | | | | 56 | | | | | | | | 0.97 (0.95-0.98) ^a^ | | | | | | | |  |
|  |  |  | Education ^a^ | | Primary | | 131 (6) | | | | | | | 61 (10) | | | | | | | | - | | | | | | | |  |
|  |  |  |  |  | High school | | 648 (31) | | | | | | | 194 (31) | | | | | | | |  |  |  |  |  |  |  |  |  |
|  |  |  |  |  | University or college | | 1289 (62) | | | | | | | 371 (59) | | | | | | | |  |  |  |  |  |  |  |  |  |
|  |  |  | Marital ^a^ status | | Married or partner | | 1559 (77) | | | | | | | 450 (72) | | | | | | | | ref | | | | | | | |  |
|  |  |  |  |  | Single | | 481 (23) | | | | | | | 173 (28) | | | | | | | | 0.66 (0.44-0.99) ^a^ | | | | | | | |  |
|  |  |  | Health | | Good or very good | | 1487 (71) | | | | | | | 456 (72) | | | | | | | | - | | | | | | | |  |
|  |  |  |  |  | Neither good nor bad | | 414 (20) | | | | | | | 128 (20) | | | | | | | |  |  |  |  |  |  |  |  |  |
|  |  |  |  |  | Bad or very bad | | 180 (9) | | | | | | | 46 (7) | | | | | | | |  |  |  |  |  |  |  |  |  |
| Kc et al. (2024) England  [38] | 1. Ecological cohort  2. GP-registered in a GP practice of more than 200 registered patients between March 23, 2020, and June 27, 2022, aged ≥15, (no N reported)  3. Univariate Negative Binomial Regressions  4. eHealth portal, EHRs, online appointment booking | Use of the NHS app for registrations, log-ins, appointments booked, prescriptions, medical record views |  | | | | Negative Binomial Regression for use  IRR (95%CI) | | | | | | | | | | | | | | | | | | | | | | | Results show strong associations between Index of Multiple Deprivation and app registration, app log-ins, appointments booked, rates of prescriptions ordered, and medical record views with lower rates in all quintiles than in Q1, lower in practices with higher proportions of male patients, and in practices with greater proportions of patients with long-term health needs. Rates were also higher in practices with higher proportions of White patients, and those with more patients from the youngest age group (aged 15–34 years). Registration rates were also higher in larger-sized practices. |
|  |  |  |  |  |  |  | Registrations | | | | Logins | | | | | Appointment booking | | | | | Medical record views | | | | Prescription ordering | | | | |  |
|  |  |  | Index of Multiple Deprivation  least deprived quintile |  | | | Ref | | | | Ref | | | | | Ref | | | | | Ref | | | | Ref | | | | |  |
|  |  |  |  | Quintile 2 | | | –9.99 ^a^ | | | | –11.04 ^a^ | | | | | –31.69 ^a^ | | | | | –8.42 ^a^ | | | | –9.05 ^a^ | | | | |  |
|  |  |  |  | Quintile 3 | | | –16.96 ^a^ | | | | –17.07 ^a^ | | | | | –48.65 ^a^ | | | | | –15.02 ^a^ | | | | –9.81 ^a^ | | | | |  |
|  |  |  |  | Quintile 4 | | | –21.63 ^a^ | | | | –20.75 ^a^ | | | | | –43.59 ^a^ | | | | | –19.43 ^a^ | | | | –7.54 ^a^ | | | | |  |
|  |  |  | Index of Multiple Deprivation most deprived quintile |  | | | –34.58 ^a^ | | | | –34.84 ^a^ | | | | | –42.74 ^a^ | | | | | –32.07 ^a^ | | | | –10.39 ^a^ | | | | |  |
|  |  |  | Sex | % Male | | | –1.46 ^a^ | | | | –2.01 ^a^ | | | | | –5.94 ^a^ | | | | | –2.45 ^a^ | | | | –2.78 ^a^ | | | | |  |
|  |  |  | Age | % youngest | | | 0.61 ^a^ | | | | 0.88 ^a^ | | | | | 1.35 ^a^ | | | | | 0.51 ^a^ | | | | –0.46 ^a^ | | | | |  |
|  |  |  | Ethnicity | % White | | | 0.30 ^a^ | | | | 0.61 ^a^ | | | | | 0.07 | | | | | 0.86 ^a^ | | | | 2.01 ^a^ | | | | |  |
|  |  |  | Health Status | % Long term illness | | | –0.28 ^a^ | | | | –0.28 ^a^ | | | | | –0.77 ^a^ | | | | | 0.10 ^a^ | | | | 0.61 ^a^ | | | | |  |
| Knöchelman et al. (2024) Germany  [41] | 1. Cross-sectional  2. Adults (≥ 18 years) from the HeReCa panel (N = 1,821)  3. Descriptive analysis, Latent Class Analysis, and Multinomial Logistic Regression  4. eHealth portal | Use of digitalized healthcare services (visit to the doctor due to out of hours, specific problem, urgent or non-urgent problem, or follow-up examination; training course on a disease; psychotherapy; prescriptions; sick note before/after examination) |  | | | | Logistic regression for use  OR CI 95%I) | | | | | | | | | | | | | | | | | | | | | | | The active users had the lowest level of education (35.6%) and were the least likely to be employed (42.9%). The proportion of women was highest among the non-users-rejecting (56.0%). An alternative operationalization of education (university entrance qualification and higher = 1, lower/no degree = 0) showed equally directed correlations for those rejecting (OR 1.23) and those actively participating (OR 0.68). In addition, there were hardly any differences in the probability of belonging to the active (OR 1.04) or the rejecting (OR 1.10) group with increasing number of previous illnesses. |
|  |  |  |  |  |  |  | Being an active user, compared to rejecting non-users | | | | | | | | | | | | | | | | | | | | | | |  |
|  |  |  | Age |  | | | 0.88 (0.71-1.08) | | | | | | | | | | | | | | | | | | | | | | |  |
|  |  |  | Sex | Male | | | Ref | | | | | | | | | | | | | | | | | | | | | | |  |
|  |  |  |  | Female | | | 0.78 (0.56-1.10) | | | | | | | | | | | | | | | | | | | | | | |  |
|  |  |  | Marital status | Single/ widowed/ divorced | | | Ref | | | | | | | | | | | | | | | | | | | | | | |  |
|  |  |  |  | Married | | | 0.77 (0.53-1.13) | | | | | | | | | | | | | | | | | | | | | | |  |
|  |  |  | Education | Less than university | | | Ref | | | | | | | | | | | | | | | | | | | | | | |  |
|  |  |  |  | University | | | 0.67 (0.47-0.95) ^a^ | | | | | | | | | | | | | | | | | | | | | | |  |
|  |  |  | Native language | German | | | Ref | | | | | | | | | | | | | | | | | | | | | | |  |
|  |  |  |  | Not German | | | 1.39 (0.67-2.86) | | | | | | | | | | | | | | | | | | | | | | |  |
|  |  |  | Employment status | Not employed | | | Ref | | | | | | | | | | | | | | | | | | | | | | |  |
|  |  |  |  | Employed | | | 0.57 (0.38-0.84) ^a^ | | | | | | | | | | | | | | | | | | | | | | |  |
|  |  |  | Place of residence | Up to 20,000 | | | Ref | | | | | | | | | | | | | | | | | | | | | | |  |
|  |  |  |  | Up to 100,000 | | | 0.96 (0.63-1.44) | | | | | | | | | | | | | | | | | | | | | | |  |
|  |  |  |  | >100,000 | | | 1.04 (0.61-1.74) | | | | | | | | | | | | | | | | | | | | | | |  |
| Pálsdóttir et al.  (2024) Iceland  [37] | 1. Prevalence study  2. Adults (≥ 18 years)  residing in Iceland (N = 3,000)  3. Descriptive  4. eHealth portal | Use of national digital healthcare system for communication with health professionals or access personal health information |  | | | | Very/rather often | | | | | | | Sometimes | | | | | | | | Seldom/never | | | | | | | | In 2019, women in the oldest group were less likely to have used it than those in the two younger groups. The use by men, was quite similar across all three age groups. There was a difference by sex. Women in the age groups 18–35 (*P* <.001) and 36–55 (*P* <.10), had used the system significantly more often than men. In 2022, the results reveal that there is a substantial increase in the use of it for both men and women in all age groups. There was a significant difference by sex in the age groups 18-35 (*P* <.001) and 36–55 (*P* <.10), with women using it more than men. |
|  |  |  |  |  |  |  | 2019 | | | 2022 | | | | 2019 | | | | 2022 | | | | 2019 | | | | 2022 | | | |  |
|  |  |  | Age and sex | 18–35 | | | % | | | % | | | | % | | | | % | | | | % | | | | % | | | |  |
|  |  |  |  | Women | | | 34.9 | | | 60.8 | | | | 12.7 | | | | 15.3 | | | | 52.4 | | | | 23.9 | | | |  |
|  |  |  |  | Men | | | 10.7 | | | 22.7 | | | | 13.6 | | | | 24.0 | | | | 75.7 | | | | 53.3 | | | |  |
|  |  |  |  | 36–55 | | |  | | |  | | | |  | | | |  | | | |  | | | |  | | | |  |
|  |  |  |  | Women | | | 33.7 | | | 49.4 | | | | 10.9 | | | | 28.3 | | | | 55.4 | | | | 22.3 | | | |  |
|  |  |  |  | Men | | | 17.9 | | | 28.1 | | | | 9.8 | | | | 33.0 | | | | 72.3 | | | | 38.8 | | | |  |
|  |  |  |  | +56 | | |  | | |  | | | |  | | | |  | | | |  | | | |  | | | |  |
|  |  |  |  | Women | | | 19.6 | | | 32.9 | | | | 9.7 | | | | 21.6 | | | | 70.7 | | | | 45.5 | | | |  |
|  |  |  |  | Men | | | 9.4 | | | 29.3 | | | | 12.9 | | | | 18.5 | | | | 78.1 | | | | 52.2 | | | |  |
| Söderberg (2024) Sweden  [33] | 1. Cross-sectional  2. Adults (≥ 18 years) residing in Sweden (N= 2,716)  3. Multivariate Logistic Regression  4. Telemedicine and Remote Primary Care | Use of digital primary care |  | | | | Physical consultation  n (%) | | | | | | | Digital consultation  n (%) | | | | | | | | Logistic regression for digital care  OR (CI 95%) | | | | | | | | Having access to internet and using internet daily were associated with seeking digital primary care (OR 5.39, CI 95% 1.17-24.77, and OR 3.21, CI 95% 1.30-7.90, respectively). Very good self-rated health, compared with average self-rated health, was also associated with seeking digital primary care (OR 1.31, CI 95% 1.01-1.69). Having university education was also associated with seeking digital care (OR 1.41, CI 95% 1.19-1.67). |
|  |  |  | Age | 18 to <38 | | | 284 (20.1) | | | | | | | 399 (30.6) | | | | | | | | Ref | | | | | | | |  |
|  |  |  |  | 38 to <51 | | | 267 (18.9) | | | | | | | 432 (33.2) | | | | | | | | 1.14 (0.91-1.43) | | | | | | | |  |
|  |  |  |  | 51 to <64 | | | 358 (25.3) | | | | | | | 334 (25.6) | | | | | | | | 0.69 (0.55-0.86) | | | | | | | |  |
|  |  |  |  | >64 | | | 507 (35.8) | | | | | | | 138 (10.6) | | | | | | | | 0.29 (0.19-0.44) | | | | | | | |  |
|  |  |  | Gender ^a^ | Male | | | 488 (34.5) | | | | | | | 386 (29.6) | | | | | | | | Ref | | | | | | | |  |
|  |  |  |  | Female | | | 919 (64.9) | | | | | | | 901 (69.1) | | | | | | | | 1.06 (0.89-1.27) | | | | | | | |  |
|  |  |  |  | Other/not specified | | | 9 (0.6) | | | | | | | 16 (1.2) | | | | | | | | 2.11 (0.86-5.19) | | | | | | | |  |
|  |  |  | Occupation^a^ | Working | | | 807 (57.5) | | | | | | | 1001 (77.4) | | | | | | | | Ref | | | | | | | |  |
|  |  |  |  | Retired | | | 451 (32.1) | | | | | | | 120 (9.3) | | | | | | | | 0.68 (0.46-1.02) | | | | | | | |  |
|  |  |  |  | Other | | | 146 (10.4) | | | | | | | 172 (13.3) | | | | | | | | 0.93 (0.72-1.21) | | | | | | | |  |
|  |  |  | Education (years) ^a^ | ≤12 or >12 | | | 641 (45.8) | | | | | | | 443 (34.3) | | | | | | | | Ref | | | | | | | |  |
|  |  |  |  | University education | | | 759 (54.2) | | | | | | | 849 (65.7) | | | | | | | | 1.41 (1.19-1.67) | | | | | | | |  |
|  |  |  | Internet habits ^a^ | Access to internet device | | | 2650 (97.6) | | | | | | | 1355 (95.8) | | | | | | | | 5.39 (1.17-24.77) | | | | | | | |  |
|  |  |  |  | Using internet daily | | | 2683 (98.8) | | | | | | | 1383 (97.8) | | | | | | | | 3.21 (1.30-7.90) | | | | | | | |  |
|  |  |  | Self-rated health ^a^ | Bad or very bad | | | 206 (7.6) | | | | | | | 104 (7.4) | | | | | | | | 1.23 (0.87-1.75) | | | | | | | |  |
|  |  |  |  | Average | | | 545 (30.1) | | | | | | | 313 (22.2) | | | | | | | | Ref | | | | | | | |  |
|  |  |  |  | Good | | | 1379 (50.9) | | | | | | | 737 (52.4) | | | | | | | | 0.99 (0.79-1.23) | | | | | | | |  |
|  |  |  |  | Very good | | | 253 (18.0) | | | | | | | 326 (25.0) | | | | | | | | 1.31 (1.01-1.69) | | | | | | | |  |
| Wilkens et al. (2024) Sweden  [39] | 1. Cross-sectional  2. Patients ≥18 (N = 726, 087)  3. Concentration index and curves; decomposition analysis; indirect standardization; horizontal inequity index.  4. Telemedicine and Remote Primary Care | Use of digital primary care |  | | | | Office-based contacts n (%) 607,586 (83,7) | | | | | | Digital contacts  n (%)  103,264 (14.2) | | | | | Both types  n (%)  15,237 (2.1) | | | | | | Decomposition of CI | | | | | | Low-income patients were more frequent users of office-based visits while high-income patients used more digital contacts. Decomposing the unequal utilization by types of primary care explained some of the inequality. For office-based visits the model specification could explain just above half of the pro-poor inequality in utilization (0.061 of the 0.116 index value), of which employment status contributes to half due to large income inequality and sensitivity to utilization. On the contrary, due to a negligible income effect, differences in age did not contribute to inequality, even though it was strongly associated with utilization of office-based visits. The large pro-rich inequality among digital contacts is explained to a smaller degree (0.083 of 0.205). High education level and being born in Sweden were the factors relatively strongly associated with the pro-rich inequality in digital contacts. For both factors, income inequality was high and there was a large sensitivity to utilization. |
|  |  |  |  |  |  |  |  |  |  |  |  |  |  |  |  |  |  |  |  |  |  |  |  | Office based | | | | Digital | |  |
|  |  |  | Contacts | Average | | | 4.4 (6.3) | | | | | | 1.5 (1.3) | | | | | 6.3 (7.0) | | | | | | Total | | | | Total | |  |
|  |  |  | Annual income | Mean | | | 212,003 (266,929) | | | | | | 294,485 (920,678) | | | | | 225,518 (200,408) | | | | | | –0.116 | | | | 0.205 | |  |
|  |  |  | Age | Mean (SD) | | | 54.0 (20.0) | | | | | | 35.9 (12.6) | | | | | 35.3 (13.8) | | | | | | –0.006 | | | | 0.019 | |  |
|  |  |  | Sex | Women | | | 341,116 (56.1) | | | | | | 67,494 (65.4) | | | | | 11,067 (72.6) | | | | | | –0.003 | | | | –0.007 | |  |
|  |  |  | Morbidity | Full health | | | 517,578 (85.2) | | | | | | 95,036 (92.0) | | | | | 14,058 (92.3) | | | | | | –0.003 | | | | 0.000 | |  |
|  |  |  |  | One diagnosis | | | 42,271 (7.0) | | | | | | 3838 (3.7) | | | | | 738 (4.8) | | | | | |  |  |  |  |  |  |  |
|  |  |  |  | Multi | | | 12,008 (2.0) | | | | | | 468 (0.5) | | | | | 100 (0.7) | | | | | |  |  |  |  |  |  |  |
|  |  |  |  | Missing | | | 35,729 (5.9) | | | | | | 3922 (3.8) | | | | | 341 (2.2) | | | | | |  |  |  |  |  |  |  |
|  |  |  | Education | Elementary | | | 138,711 (22.8) | | | | | | 8934 (8.7) | | | | | 1702 (11.2) | | | | | | –0.013 | | | | 0.023 | |  |
|  |  |  |  | High school | | | 275,738 (45.4) | | | | | | 39,665 (38.4) | | | | | 7006 (46.0) | | | | | |  |  |  |  |  |  |  |
|  |  |  |  | University | | | 184,232 (30.3) | | | | | | 5397 (52.3) | | | | | 6473 (42.5) | | | | | |  |  |  |  |  |  |  |
|  |  |  |  | Missing | | | 8905 (1.5) | | | | | | 695 (0.7) | | | | | 56 (0.4) | | | | | |  |  |  |  |  |  |  |
|  |  |  | Country of birth | Foreign | | | 111,077 (18.3) | | | | | | 15,132 (14.7) | | | | | 1675 (11.0) | | | | | | –0.003 | | | | 0.022 | |  |
|  |  |  |  | Sweden | | | 496,509 (81.7) | | | | | | 88,132 (85.3) | | | | | 13,562 (89.0) | | | | | |  |  |  |  |  |  |  |
|  |  |  | Geographic region | Rural | | | 141,804 (23.3) | | | | | | 4976 (4.8) | | | | | 2467 (16.2) | | | | | | –0.003 | | | | 0.013 | |  |
|  |  |  |  | Sub-urban | | | 20,207 (33.3) | | | | | | 28,547 (27.6) | | | | | 4068 (26.7) | | | | | |  |  |  |  |  |  |  |
|  |  |  |  | Urban | | | 263,712 (43.4) | | | | | | 69,741 (67.5) | | | | | 8702 (57.1) | | | | | |  |  |  |  |  |  |  |
|  |  |  | Employment status | Non-employed | | | 276,990 (45.6) | | | | | | 14,720 (14.3) | | | | | 2955 (19.4) | | | | | | –0.032 | | | | 0.013 | |  |
|  |  |  |  | Employed | | | 330,596 (54.4) | | | | | | 88,544 (85.7) | | | | | 12,282 (80.6) | | | | | |  |  |  |  |  |  |  |
| Zhang et al. (2023) England  [46] | 1. Cross-sectional  2. Populations of primary  care practices in National Health Service  3. Multivariate Linear Regression  4. eHealth portal | Use of National Health service app and a primary care portal. |  | | | | Linear regression for use | | | | | | | | | | | | | | | | | | | | | | | Increased population from the two most socioeconomically deprived quintiles was associated with reduced NHS App activation (quintile 1: coef −0.223, CI 97.5% −0.232 to −0.213, *P* <0.001; quintile 2: coef −0.117, CI 97.5% −0.128 to −0.106, *P* <0.001). The least deprived quintile was associated with greater activation (coef 0.121, 97.5% CI 0.111-0.131, *P* <.001). Other notable associations were seen with age (76–85 years: coef −0.177, CI 97.5% −0.312 to −0.041, *P* <.05) and urbanity/rurality (urban: coef 0.043, CI 97.5% 0.037-0.049, p<.001). Similar findings were found in primary care portals, with negative association of deprived quintiles (quintile 1: coef −2.047, 97.5% CI−2.247 to −1.847, *P* <.001; quintile 2: coef −1.114, 97.5% CI −1.348 to −0.880, *P* <.001), and positive association with the least deprived (coef 1.269, CI 97.5% 1.055 to 1.482, *P* <.001). Directional associations across age and urbanity/rurality were preserved. |
|  |  |  |  |  |  |  | National Health Service app activated | | | | | | | | | | | Linear regression for use | | | | | | | | | | | |  |
|  |  |  |  |  |  |  | Univariate | | | | | | Multivariable | | | | | Univariate | | | | | | Multivariable | | | | | |  |
|  |  |  | Deprivation | | Q1 | | –0.248^a^ | | | | | | –0.223^a^ | | | | | –2.338^a^ | | | | | | –2.047^a^ | | | | | |  |
|  |  |  |  | | Q3 | | –0.181^a^ | | | | | | –0.117^a^ | | | | | –1.625^a^ | | | | | | –1.114^a^ | | | | | |  |
|  |  |  |  | | Q5 | | 0.281^a^ | | | | | | 0.121^a^ | | | | | 2.567^a^ | | | | | | 1.269^a^ | | | | | |  |
|  |  |  | Age | | 16–25 | | 0.004 | | | | | | 0.293^a^ | | | | | –1.842^a^ | | | | | | 0.184 | | | | | |  |
|  |  |  |  | | 26–35 | | –0.310^a^ | | | | | | 0.241^a^ | | | | | –2.225^a^ | | | | | | 5.819^a^ | | | | | |  |
|  |  |  |  | | 56–65 | | 0.685^a^ | | | | | | 0.585^a^ | | | | | 8.491^a^ | | | | | | 8.872^a^ | | | | | |  |
|  |  |  |  | | 76–85 | | 1.027^a^ | | | | | | –0.177^a^ | | | | | 10.832^a^ | | | | | | –0.889 | | | | | |  |
|  |  |  | Ethnicity | | Black | | –0.312^a^ | | | | | | –016 | | | | | –3.017^a^ | | | | | | 0.034 | | | | | |  |
|  |  |  |  |  | Asian | | –0.172^a^ | | | | | | 0.016 | | | | | –1.469^a^ | | | | | | 0.973^a^ | | | | | |  |
|  |  |  | Residence | | Urban | | –0.048^a^ | | | | | | 0.043^a^ | | | | | –0.538^a^ | | | | | | 0.298^a^ | | | | | |  |
|  |  |  | Any long-term condition | |  | | –0.157^a^ | | | | | | –0.091^a^ | | | | | –1.111^a^ | | | | | | –1.161^a^ | | | | | |  |
| Pierce et al. (2023) England  [47] | 1. Cross-sectional  2. Pregnant women booked into UCLH  for their initial antenatal appointment in February 2022, identified  through the EPIC platform (N = 636)  3. Descriptive  4. eHealth portal | Use of MyCare, an electronic patient portal (access to test results, information about appointments, and  enables communication with healthcare professionals) |  | | | | Downloaded  n=597 (93.9%) | | | | | | | | | | | | | | | Not downloaded  n=39 (6.1%)  Category 3 | | | | | | | | Category 3 had a lower average age (30). There was a link between a higher average parity and lower engagement or non-use, with the average parity being 0.94, 1.47, and 1.78 in categories 1, 2, and 3 respectively. There was an association between higher rates of vulnerability and lower engagement or non-use, with the percentage of vulnerable individuals being 19.7%, 26%, and 48.7% in categories 1, 2, and 3 respectively. Mental health issues, alongside physical and learning disabilities, were the most prevalent vulnerabilities within the low engagement category. Mental health issues, domestic violence, and refugee/asylum seeker status were the most prevalent vulnerabilities in the group who did not have MyCare. The percentage of women whose first language was not English was considerably higher for those without MyCare (48.7%).  For Category 3 the average Social Deprivation Index decile was lower (3.72). |
|  |  |  |  |  |  |  | >50% Engagement (87.8%)  Category 1 | | | | | | | <50% Engagement (12.2%)  Category 2 | | | | | | | |  |  |  |  |  |  |  |  |  |
|  |  |  | Age | Average | | | 33 | | | | | | | 34 | | | | | | | | 30 | | | | | | | |  |
|  |  |  | Parity | Average | | | 0.94 | | | | | | | 1.47 | | | | | | | | 1.78 | | | | | | | |  |
|  |  |  | Vulnerable | | | | 19.7% | | | | | | | 26.0% | | | | | | | | 48.7% | | | | | | | |  |
|  |  |  | English not first language | | | | 24.4% | | | | | | | 23.3% | | | | | | | | 48.7% | | | | | | | |  |
|  |  |  | Social deprivation index decile | Average | | | 4.75 | | | | | | | 4.76 | | | | | | | | 3.72 | | | | | | | |  |
| Heponiemi et al.  (2022)  Finland  [45] | 1. Cross-sectional  2. Sample from the Population Register of Finland  >20years  (N = 4,495)  3. Multivariate Logistic Regression  4. EHRs, eHealth portal, online appointment booking | Use of online health services (receiving test results, requesting a  renewal of a prescription, scheduling an  appointment, and having an appointment with a  health or social care professional) |  | | | | Logistic regression for use | | | | | | | | | | | | | | | | | | | | | | | While having an online appointment was very uncommon, the use of other online services was relatively frequent. Age was linked to all types of online service use except for having an online appointment. Relationship between age and online service use showed a steeper decline among older adults starting around age 60. These age-related associations remained statistically significant even after adjusting for digital competence. Overall, the association between good (versus poor) digital competence and online service use was strong: OR 12.61 (CI 95% 8.52-18.64) for receiving lab or other test results, OR 8.82 (CI 95% 6.15-12.64) for prescription renewal, OR 10.91 (CI 95% 7.24-16.44) for scheduling an appointment, and OR 6.48 (CI 95% 0.93-45.12, not significant) for having an appointment. |
|  |  |  |  |  |  |  | Received Test Results  (n=2403) | | | | | | Renewed a Prescription (n=2190) | | | | | Scheduled an appointment  (n=2003) | | | | | | Had an appointment  (n=2271) | | | | | |  |
|  |  |  |  |  |  |  |  | OR | | aOR^e^ | | |  | OR | | | aOR |  | | OR | | aOR | |  | | OR | | | aOR |  |
|  |  |  | Age | | Mean | | 53.2 | 0.14 ^a^ | | 0.15 ^a^ | | | - | 0.03 ^a^ | | | 0.02 | - | | 0.48 | | 0.45 | | - | | 0.01 | | | 1.01 |  |
|  |  |  | Age^2 | |  | | - | 1.69 ^a^ | | 1.66 ^a^ | | | - | 2.25 ^a^ | | | 2.38 | - | | 1.35 | | 1.38 | | - | | 3.07 | | | 3.02 |  |
|  |  |  | Age^3 | |  | | - | 0.96 ^a^ | | 0.96 ^a^ | | | - | 0.94 ^a^ | | | 0.94 | - | | 0.97 ^a^ | | 0.97 ^a^ | | - | | 0.92 | | | 0.92 |  |
|  |  |  | Gender | | Female | | 58% | - | | - | | | 58% | - | | | - | 58% | | - | | - | | 59% | | - | | | - |  |
|  |  |  | Digital competence | | Poor | | 21% | - | | - | | | 25% | - | | | - | 17% | | - | | - | | 18% | | - | | | - |  |
|  |  |  |  |  | Average | | 24% | - | | 5.86 | | | 25% | - | | | 4.23 ^a^ | 23% | | - | | 4.86 ^a^ | | 23% | | - | | | 1.16 |  |
|  |  |  |  |  | Good | | 55% | - | | 12.6 | | | 49% | - | | | 8.82 ^a^ | 60% | | - | | 10.9 ^a^ | | 59% | | - | | | 6.48 |  |
|  |  |  | Online use | | Yes | | 47% | - | | - | | | 43% | - | | | - | 54% | | - | | - | | 2% | | - | | | - |  |
|  |  |  |  |  | No | | 53% | - | | - | | | 57% | - | | | - | 46% | | - | | - | | 98% | | - | | | - |  |
| Chapman et al.  (2022)  England  [43] | 1. Cross-sectional  2. Adults ≥18 receiving hospital outpatient care  (N = 28,637)  3. Descriptive, Univariate and Multivariate Logistic Regression  4. EHRs | Sign up and activation of “myHealth @QEHB”, a hospital-based Personal Health Record |  | | | | No signed up n=20567  n (%) | | Signed up but not activated n=3784  n (%) | | | Activated  n=4286  n (%) | | | Logistic regression for sign up and activation | | | | | | | | | | | | | | | Males were less likely than females to activate their Personal Health Record (aOR 0.85, CI 95% 0.78- 0.94).In contrast,sign up was more likely in males (aOR 1.10). Differences were also seen by age with patients aged 35–54 being more likely than other age groups. While those aged 16–34 had been more likely to be signed up, they were less likely to activate their Personal Health Record compared with those aged 35–54 (aOR 0.80, CI 95% 0.70-0.91). Aged 75 and older were the least likely to do so (aOR 0.39, CI 95% 0.32-0.47). The pattern of increasing likelihood of sign up among patients from less deprived areas was also seen with the likelihood of activation, with those in the least deprived areas again being three times more likely to activate their accounts than those from the most deprived areas (aOR 2.99, CI 95% 2.40-3.71).  Patients from Asian, black and mixed ethnic groups were all significantly less likely than those from White ethnic groups to activate their Personal Health Record accounts. Similarly, patients who did not need an interpreter were over three times more likely to activate their accounts than those who did need one (aOR 3.16, CI 95% 1.96-5.09). While patients who were registered with more than one clinical specialty were more likely to be signed up, there was no significant association between the number of specialties and the likelihood of activating. |
|  |  |  |  |  |  |  |  |  |  |  |  |  |  |  | Likelihood of sing up | | | | | | | | Likelihood of activation among those who had been signed up | | | | | | |  |
|  |  |  |  |  |  |  |  |  |  |  |  |  |  |  | OR  (CI 95%) | | | | aOR  (CI 95%) | | | | OR  (CI 95%) | | | | aOR  (CI 95%) | | |  |
|  |  |  | Gender | | | Females | 10,837 (52.7) | | 1813 (47.9) | | | 2,064 48.2 | | | Ref | | | | Ref | | | | Ref | | | | Ref | | |  |
|  |  |  |  | | | Males | 9,725 (47.3) | | 1971 (13.2) | | | 2,222 (51.8) | | | 1.11  (1.06-1.17) | | | | 1.10  (1.04-1.16) | | | | 0.85  (0.78-0.93) ^a^ | | | | 0.85  (0.7-0.94) ^a^ | | |  |
|  |  |  |  | | | Unknown | 5 (<1) | | 0 | | | 0 | | |  | | | |  | | | | - | | | | - | | |  |
|  |  |  | Age | | | 16-34 | 3442 (16.7) | | 820 (21.7) | | | 879 (20.5) | | | 1.25  (1.17-1.35) | | | | 1.47  (1.38-1.61) | | | | 0.83  (0.73-0.94) ^a^ | | | | 0.80  (0.70-0.91) ^a^ | | |  |
|  |  |  |  | | | 35-54 | 6646 (32.3) | | 1136 (30) | | | 1471 (34.3) | | | Ref | | | | Ref | | | | Ref | | | | Ref | | |  |
|  |  |  |  | | | 55-74 | 7440 (36.2) | | 1433 (37.9) | | | 1735 (40.5) | | | 1.08  (1.02-1.15) | | | | 0.94  (0.88-1.00) | | | | 0.94  (0.84- 1.04) | | | | 0.86  (0.77-0.96) | | |  |
|  |  |  |  | | | 75+ | 3039 (14.8) | | 395 (10.4) | | | 201 (4.7) | | | 0.50  (0.45-0.55) | | | | 0.40  (0.36-0.44) | | | | 0.39  (0.33-0.47) ^a^ | | | | 0.39  (0.32-0.47) ^a^ | | |  |
|  |  |  | Deprivation Decile | | | 1 | 5296 (26.3) | | 982 (26.5) | | | 582 (14.3) | | | Ref | | | | Ref | | | | Ref | | | | Ref | | |  |
|  |  |  |  |  |  | 2 | 3116 (15.5) | | 550 (14.8) | | | 438 (10.7) | | | 1.07  (0.98-1.18) | | | | 1.10  (1.00-1.20) | | | | 1.34  (1.14-1.58) ^a^ | | | | 1.21  (1.03-1.43) | | |  |
|  |  |  |  |  |  | 3 | 2108 (10.5) | | 386 (10.3) | | | 397 (9.7) | | | 1.25  (1.13-1.38) | | | | 1.26  (1.14-1.40) | | | | 1.75  (1.47-2.08) ^a^ | | | | 1.50  (1.25-1.79) ^a^ | | |  |
|  |  |  |  |  |  | 4 | 1693 (8.4) | | 316 (8.5) | | | 395 (9.7) | | | 1.42  (1.28-1.58) | | | | 1.46  (1.31-1.63) | | | | 2.11  (1.76-2.53) ^a^ | | | | 1.79  (1.49-2.16) ^a^ | | |  |
|  |  |  |  |  |  | 5 | 2146 (10.6) | | 427 (11.5) | | | 470 (11.5) | | | 1.41  (1.28-1.56) | | | | 1.46  (1.32-1.61) | | | | 1.86  (1.57-2.19) ^a^ | | | | 1.63 (1.38-1.94) ^a^ | | |  |
|  |  |  |  |  |  | 6 | 1431 (7.1) | | 280 (7.6) | | | 371 (9.1) | | | 1.54  (1.38-1.72) | | | | 1.67  (1.49-1.87) | | | | 2.23  (1.86-2.69) ^a^ | | | | 1.84  (1.52-2.23) ^a^ | | |  |
|  |  |  |  |  |  | 7 | 1394 (6.9) | | 238 (6.4) | | | 358 (8.8) | | | 1.45  (1.30-1.62) | | | | 1.57  (1.40-1.77) | | | | 2.54  (2.09-3.08) ^a^ | | | | 2.06  (1.69-2.52) ^a^ | | |  |
|  |  |  |  |  |  | 8 | 1106 (5.5) | | 188 (5.1) | | | 351 (8.6) | | | 1.65  (1.47-1.86) | | | | 1.87  (1.66-2.12) | | | | 3.15  (2.57-3.87) ^a^ | | | | 2.53  (2.05-3.13) ^a^ | | |  |
|  |  |  |  |  |  | 9 | 948 (4.7) | | 172 (4.6) | | | 355 (8.7) | | | 1.88  (1.67-2.12) | | | | 2.15  (1.90-2.44) | | | | 3.48  (2.83-4.29) ^a^ | | | | 2.74  (2.21-3.40) ^a^ | | |  |
|  |  |  |  |  |  | 10 | 910 (4.5) | | 169 (4.6) | | | 361 (8.8) | | | 1.97  (1.75-2.23) | | | | 2.31  (2.04-2.63) | | | | 3.60  (2.92-4.44) ^a^ | | | | 2.99  (2.40-3.71) ^a^ | | |  |
|  |  |  | Ethnicity | | | White | 10 116 (49.2) | | 10116  (49.2) | | | 2780 (64.9) | | | Ref | | | | Ref | | | | Ref | | | | Ref | | |  |
|  |  |  |  |  |  | Asian | 2567 (12.5) | | 2567  (12.5) | | | 404 (9.4) | | | 0.85  (0.79-0.93) | | | | 0.98  (0.90-1.06) | | | | 0.46  (0.40-0.53) ^a^ | | | | 0.61  (0.53-0.71) ^a^ | | |  |
|  |  |  |  |  |  | Black | 1075 (5.2) | | 1075  (5.2) | | | 130 (3.0) | | | 0.80  (0.71-0.90) | | | | 0.98  (0.86- 1.11) | | | | 0.34  (0.27-0.42) ^a^ | | | | 0.45  (0.36-0.56) ^a^ | | |  |
|  |  |  |  |  |  | Mixed | 271 (1.3) | | 271  (1.3) | | | 147 (3.4) | | | 2.38  (2.02- 2.81) | | | | 2.62  (2.20-3.12) | | | | 0.67  (0.53-0.84) ^a^ | | | | 0.77  (0.60-0.97) ^a^ | | |  |
|  |  |  |  |  |  | Other | 542 (2.6) | | 542  (2.6) | | | 75 (1.8) | | | 0.64  (0.53-0.76) | | | | 0.46  (0.43-0.49) | | | | 0.61  (0.45-0.84) ^a^ | | | | 0.75  (0.54-1.05) | | |  |
|  |  |  |  |  |  | Not known | 5996 (29.2) | | 5996  (29.2) | | | 750 (17.5) | | | 0.47  (0.44-0.51) | | | | 0.75  (0.62-0.90) | | | | 0.92  (0.81-1.04) | | | | 0.88  (0.77-1.00) | | |  |
|  |  |  | Need an interpreter | | | Yes | 722 (3.5) | | 722  (3.5) | | | 22 (0.5) | | | Ref | | | | Ref | | | | Ref | | | | Ref | | |  |
|  |  |  |  |  |  | No | 19 845 (96.5) | | 19845  (96.5) | | | 4264 (99.5) | | | 2.24  (1.85-2.71) | | | | 1.63  (1.33-1.99) | | | | 5.64  (3.56-8.94) ^a^ | | | | 3.16  (1.96-5.09) ^a^ | | |  |
|  |  |  | Number of hospital specialties | | | 1 | 9,957 (48.4) | | 9957  (48.4) | | | 1873 (43.7) | | | Ref | | | | Ref | | | | Ref | | | | Ref | | |  |
|  |  |  |  |  |  | 2 to 4 | 9433 (45.9) | | 9433  (45.9) | | | 1960 (45.7) | | | 1.26  (1.19-1.33) | | | | 1.32  (1.24-1.40) | | | | 0.75  (0.68-0.82) ^a^ | | | | 0.88  (0.79-0.97) ^a^ | | |  |
|  |  |  |  |  |  | 5 + | 1177 (5.7) | | 1177  (5.7) | | | 453 (10.6) | | | 2.30  (2.09-2.54) | | | | 2.54  (2.30-2.82) | | | | 0.77  (0.66-0.90) ^a^ | | | | 1.01  (0.86-1.18) | | |  |
| Neves et al.  (2021)  United Kingdom  [42] | 1. Cross-sectional  2. Patients >18 of hospitals and primary care in London, registeredin the Care Information Exchange  (N = 650)  3. Descriptive, Univariate and Multivariate Logistic Regression  4. eHealth portal | Use of Care Information Exchange, a patient portal containing patient information (appointment details, test results, care plans, discharge  summaries, clinical letters, and information on medications) |  | | | | Non-users n = 205  n (%) | | | | | | Users n = 447  n (%) | | | | | Logistic Regression for use | | | | | | | | | | | | The proportion of users was 61.7%.  Crude ORs showed that individuals with a higher educational degree (undergraduate/professional or postgraduate/higher) had higher odds of being portal users (1.48, CI 95% 1.00-2.20 and 2.15, CI 95% 1.33-3.05, respectively). Higher digital literacy scores (>30) were associated with greater odds (2.90, CI 95%: 2.06-4.11). Good health status was associated with lower odds (0.63, CI 95% 0.43-0.94). Adjusted ORs confirmed these associations: individuals with higher educational degree had increased odds of portal use (1.58, CI 95% 1.04-2.39 and 2.38, CI 95% 1.42-4.02); higher digital literacy (≥30) was also associated (2.96, CI 95% 2.02-4.35); Those with a good overall health status had lower odds of being a user (adjusted OR 0.58, CI 95% 0.37-0.91). |
|  |  |  |  |  |  |  |  |  |  |  |  |  |  |  |  |  |  | OR (CI 95%) | | | | | | aOR (CI 95%) | | | | | |  |
|  |  |  | Gender | | | Female | 113 (55.1) | | | | | | 276 (61.7) | | | | | Ref | | | | | | Ref | | | | | |  |
|  |  |  |  |  |  | Male | 91 (44.4) | | | | | | 167 (37.4) | | | | | 0.75 (0.54-1.05) | | | | | | 0.92 (0.624-1.35) | | | | | |  |
|  |  |  |  |  |  | Other | 1 (0.5) | | | | | | 2 (0.4) | | | | | 0.81 (0.07-9.12) | | | | | | 0 (0-infinity) | | | | | |  |
|  |  |  | Age | | | <30 | 9 (4.4) | | | | | | 22 (4.9) | | | | | Ref | | | | | | Ref | | | | | |  |
|  |  |  |  |  |  | 31-40 | 20 (9.8) | | | | | | 48 (10.7) | | | | | 0.98 (0.39-2.50) | | | | | | 0.63 (0.22-1.76) | | | | | |  |
|  |  |  |  |  |  | 41-50 | 23 (11.2) | | | | | | 62 (13.9) | | | | | 1.10 (0.44-2.74) | | | | | | 0.88 (0.32-2.40) | | | | | |  |
|  |  |  |  |  |  | 51-65 | 72 (35.1) | | | | | | 166 (37.1) | | | | | 0.94 (0.41-2.15) | | | | | | 0.85 (0.34-2.12) | | | | | |  |
|  |  |  |  |  |  | ≥65 | 81 (39.5) | | | | | | 147 (32.9) | | | | | 0.74 (0.33-1.69) | | | | | | 0.65 (0.26-1.65) | | | | | |  |
|  |  |  | Ethnicity | | | White | 155 (75.6) | | | | | | 343 (76.7) | | | | | Ref | | | | | | - | | | | | |  |
|  |  |  |  |  |  | Black,Asian,minority ethnic | 34 (16.6) | | | | | | 75 (16.8) | | | | | 0.88 (0.59-1.33) | | | | | | - | | | | | |  |
|  |  |  |  |  |  | Other | 16 (7.8) | | | | | | 22 (4.9) | | | | | - | | | | | | - | | | | | |  |
|  |  |  | Education | | | Secondary school | 75 (36.6) | | | | | | 118 (61.1) | | | | | Ref | | | | | | Ref | | | | | |  |
|  |  |  |  |  |  | Under-graduate | 77 (37.6) | | | | | | 180 (40.3) | | | | | 1.48 (1.00-2.20) ^a^ | | | | | | 1.58 (1.04-2.39) ^a^ | | | | | |  |
|  |  |  |  |  |  | Post-graduate | 33 (16.1) | | | | | | 112 (25.1) | | | | | 2.15 (1.33-3.50) ^a^ | | | | | | 2.38 (1.42-4.02) ^a^ | | | | | |  |
|  |  |  | Health  status | | | Poor or very poor | 55 (26.8) | | | | | | 162 (36.2) | | | | | Ref | | | | | | Ref | | | | | |  |
|  |  |  |  |  |  | Neither good nor poor | 55 (26.8) | | | | | | 106 (23.7) | | | | | 0.65 (0.42-1.02) | | | | | | 0.73 (0.45-1.20) | | | | | |  |
|  |  |  |  |  |  | Good or very good | 95 (46.3) | | | | | | 177 (39.6) | | | | | 0.63 (0.43-0.94) ^a^ | | | | | | 0.58 (0.37-0.91) ^a^ | | | | | |  |
|  |  |  | eHEALS score | | | Mean (SD) | 28.4 (8.1) | | | | | | 32.9 (7.4) | | | | | - | | | | | | - | | | | | |  |
|  |  |  |  |  |  | Score<30 | - | | | | | | - | | | | | Ref | | | | | | Ref | | | | | |  |
|  |  |  |  |  |  | Score>30 | - | | | | | | - | | | | | 2.90 (2.06-4.11) ^a^ | | | | | | 2.96 (2.02-4.35) ^a^ | | | | | |  |
| Dahlgren  et al.  (2021)  Sweden  [44] | 1. Cross-Sectional  2. Adults (≥18 years) residing in Stockholm County and registered with a publicly funded PHC provider  (N = 1,991,995)  3. Descriptive and Multivariate Logistic Regression  4. Telemedicine and Remote Primary Care | Use of telemedicine to provide traditional primary care |  | | | | Logistic regression for use | | | | | | | | | | |  | | | | | | | | | | | | Although the number of telemedicine consultations has increased substantially since the introduction in 2016, the number of consultations was still low in comparison to physician office visits in primary care in 2018. Patient factors that increased the likelihood of having made at least one Direct To Consumer telemedicine consultation in 2018 were: being a woman, being of younger age, being born in Sweden, having a higher educational attainment and income and having had a history of depression or COPD/asthma.  Factors that increased the likelihood of having made a physician office visit differed to a great extent from the Direct To Consumer telemedicine consultations. A history of heart failure and diabetes, being born outside of EU28, having a lower educational attainment, a lower level of income, and being in the age group 65 + were factors that increased the likelihood of making a physician office visits but decreased the likelihood of making a telemedicine consultation. |
|  |  |  |  |  |  |  | Face-to-face physician office visit | | | | | | | | | | | Direct-to-consumer telemedicine physician consultations | | | | | | | | | | | |  |
|  |  |  |  |  |  |  | n (%) | | | | | | OR (CI 95%) | | | | | n (%) | | | | | | OR (CI 95%) | | | | | |  |
|  |  |  | Gender | Men | | | 484,292 (49.5) | | | | | | Ref | | | | | 49,769 (5.1) | | | | | | Ref | | | | | |  |
|  |  |  |  | Women | | | 602,486 (595) | | | | | | 1.46 (1.45-1.47) ^a^ | | | | | 76,198 (7.5) | | | | | | 1.60 (1.58-1.62) ^a^ | | | | | |  |
|  |  |  | Age | 19-25 | | | 64,470 (44.3) | | | | | | Ref | | | | | 14,477 (9.9) | | | | | | Ref | | | | | |  |
|  |  |  |  | 26-45 | | | 283,483 (48.7) | | | | | | 1.15 (1.13-1.16) ^a^ | | | | | 45,962 (7.9) | | | | | | 0.82 (0.81-0.84) ^a^ | | | | | |  |
|  |  |  |  | 46-64 | | | 284,019 (58.5) | | | | | | 1.69 (1.69-1.71) ^a^ | | | | | 15,836 (3.3) | | | | | | 0.30 (0.29-0.31) ^a^ | | | | | |  |
|  |  |  |  | 65+ | | | 250,429 (78.0) | | | | | | 3.81 (3.76-3.87) ^a^ | | | | | 2159 (0.7) | | | | | | 0.07 (0.06-0.07) ^a^ | | | | | |  |
|  |  |  | Highest completed level of education | Lower secondary | | | 133,272 (63.7) | | | | | | Ref | | | | | 5336 (2.5) | | | | | | Ref | | | | | |  |
|  |  |  |  | Upper secondary | | | 399,397 (56.9) | | | | | | 0.97 (0.96-0.98) ^a^ | | | | | 38,487 (5.5) | | | | | | 1.34 (1.30-1.38) ^a^ | | | | | |  |
|  |  |  |  | Post-secondary less than 3 years | | | 184,977 (53.8) | | | | | | 0.94 (0.93-0.95) ^a^ | | | | | 24,713 (7.2) | | | | | | 1.51 (1.47-1.56) ^a^ | | | | | |  |
|  |  |  |  | Post-secondary 3 years or more | | | 369,132 (50.1) | | | | | | 0.86 (0.85-0.87) ^a^ | | | | | 57,431 (7.8) | | | | | | 1.38 (1.34-1.42) ^a^ | | | | | |  |
|  |  |  | Country of birth | Sweden | | | 836,574 (53.4) | | | | | |  | | | | | 112,658 (7.2) | | | | | | Ref | | | | | |  |
|  |  |  |  | EU28^g^ | | | 80,429 (59.1) | | | | | | Ref | | | | | 4185 (3.1) | | | | | | 0.63 (0.61-0.65) ^a^ | | | | | |  |
|  |  |  |  | Outside EU28 | | | 169,775 (59.0) | | | | | | 1.00 (0.99-1.01) | | | | | 9124 (3.2) | | | | | | 0.53 (0.52-0.55) ^a^ | | | | | |  |
|  |  |  | Income  Group | 1 | | | 77,198 (51.2) | | | | | | 0.31 (1.30-1.32) ^a^ | | | | | 6097 (4.0) | | | | | | Ref | | | | | |  |
|  |  |  |  | 2 | | | 116,459 (60.9) | | | | | | Ref | | | | | 7978 (4.2) | | | | | | 1.19 (1.15-1.24) ^a^ | | | | | |  |
|  |  |  |  | 3 | | | 121,857 (59.6) | | | | | | 1.20 (1.18-1.22) ^a^ | | | | | 10,884 (5.3) | | | | | | 1.42 (1.37–-146) ^a^ | | | | | |  |
|  |  |  |  | 4 | | | 121,345 (57.8) | | | | | | 1.24 (1.22-1.26) ^a^ | | | | | 13,083 (6.2) | | | | | | 1.58 (1.531.63) ^a^ | | | | | |  |
|  |  |  |  | 5 | | | 117,813 (55.6) | | | | | | 1.25 (1.23-1.27) ^a^ | | | | | 14,676 (6.9) | | | | | | 1.72 (1.67-1.78) ^a^ | | | | | |  |
|  |  |  |  | 6 | | | 113,401 (54.0) | | | | | | 1.21 (1.20-1.23) ^a^ | | | | | 14,747 (7.0) | | | | | | 1.77 (1.71-1.83) ^a^ | | | | | |  |
|  |  |  |  | 7 | | | 110,572 (52.8) | | | | | | 1.17 (1.15-1.19) ^a^ | | | | | 14,807 (7.1) | | | | | | 1.86 (1.80-1.92) ^a^ | | | | | |  |
|  |  |  |  | 8 | | | 107,106 (52.2) | | | | | | 1.11 (1.10-1.13) ^a^ | | | | | 14,532 (7.1) | | | | | | 1.99 (1.93-2.06) ^a^ | | | | | |  |
|  |  |  |  | 9 | | | 103,682 (51.5) | | | | | | 1.07 (1.05-1.09) ^a^ | | | | | 14,001 (6.9) | | | | | | 2.11 (2.05-2.18) ^a^ | | | | | |  |
|  |  |  |  | 10 | | | 97,345 (49.2) | | | | | | 1.01 (1.99-1.02) ^a^ | | | | | 15,162 (7.7) | | | | | | 2.46 (2.38-2.54) ^a^ | | | | | |  |
|  |  |  | Diagnoses for chronic conditions | No Heart failure | | | 1,070,560 (54.3) | | | | | | 0.89 (1.87-0.90) | | | | | 125,864 (6.4) | | | | | | Ref | | | | | |  |
|  |  |  |  | heart failure | | | 16,218 (82.8) | | | | | | Ref | | | | | 103 (0.5) | | | | | | 0.49 (0.40-0.59) ^a^ | | | | | |  |
|  |  |  |  | No Depression | | | 977,349 (53.1) | | | | | | 1.45 (1.4-1.51) ^a^ | | | | | 114,291 (6.2) | | | | | | Ref | | | | | |  |
|  |  |  |  | depression | | | 109,429 (72.6) | | | | | | Ref | | | | | 11,676 (7.8) | | | | | | 1.47 (1.44-1.50) ^a^ | | | | | |  |
|  |  |  |  | No Diabetes | | | 1,012,655 (53.2) | | | | | | 2.17 (2.14-2.19) ^a^ | | | | | 124,560 (6.5) | | | | | | Ref | | | | | |  |
|  |  |  |  | diabetes | | | 74,123 (84.1) | | | | | | Ref | | | | | 1407 (1.6) | | | | | | 0.76 (0.72-0.80) ^a^ | | | | | |  |
|  |  |  |  | No COPD^f^/asthma | | | 973,676(53.3) | | | | | | 2.75 (1.70-2.81) ^a^ | | | | | 112,777 (6.2) | | | | | | Ref | | | | | |  |
|  |  |  |  | COPD/asthma | | | 113,102(68.9) | | | | | | Ref | | | | | 13,190 (8.0) | | | | | | 1.37 (1.341.40) ^a^ | | | | | |  |
|  |  |  | Primary care  accessibility | Low | | | 333,301 (51.4) | | | | | | 1.79 (1.76-1.81) ^a^ | | | | | 41,747 (6.4) | | | | | | Ref | | | | | |  |
|  |  |  |  | Medium | | | 387,337 (55.0) | | | | | | Ref | | | | | 44,762 (6.4) | | | | | | 0.97 (0.96-0.98) ^a^ | | | | | |  |
|  |  |  |  | High | | | 366,140 (57.3) | | | | | | 1.20 (1.20-1.21) ^a^ | | | | | 39,458 (6.2) | | | | | | 088 (0.87-0.90) ^a^ | | | | | |  |
|  |  |  | Distance to chosen primary healthcare centre | 0-1 km | | | 442,134 (56.8) | | | | | | 1.37 (1.36-1.38) ^a^ | | | | | 44,973 (5.8) | | | | | | Ref | | | | | |  |
|  |  |  |  | 1-2 km | | | 250,164 (55.0) | | | | | | Ref | | | | | 29,831 (6.6) | | | | | | 1.05 (1.04-1.07) ^a^ | | | | | |  |
|  |  |  |  | 2-4 km | | | 183,248 (53.7) | | | | | | 0.97 (0.97-0.98) ^a^ | | | | | 24,294 (7.1) | | | | | | 1.11 (1.09-1.13) ^a^ | | | | | |  |
|  |  |  |  | 4-10 km | | | 127,241 (51.8) | | | | | | 0.95 (0.94-0.96) ^a^ | | | | | 16,848 (6.9) | | | | | | 1.09 (1.07-1.11) ^a^ | | | | | |  |
|  |  |  |  | 10+ km | | | 83,991 (48.8) | | | | | | 0.87 (0.87-0.88) ^a^ | | | | | 10,021 (5.8) | | | | | | 1.02 (1.00-1.05) | | | | | |  |
| Merkel et al.  (2020)  Europe  [40] | 1. Cross–sectional  2. Adults aged >65 that use internet (N = 6900)  3. Multilevel Logistic Regression  4. eHealth portal | Use of internet-based health care services (any service, not specified) |  | | | | Nonusers  n =5787  n (%) | | | | | | | Users  n=1113  n (%) | | | | | | | | Logistic Regression for use  OR (CI 95%) | | | | | | | | Users were younger and better educated. Among those from a high social class an those with a partner, there was a higher proportion of users than nonusers. Among those in one-person households, there was a lower proportion of users than nonusers.  Higher age was associated with lower likelihood of using eHealth (OR 0.97, CI 95% 0.96-0.98, *P* <.001). Significant positive associations were found between education (16–19 years: OR 1.43, CI 95% 1.15–2.79; ≥20 years: OR 1.95, CI 95% 1.54–2.46; *P* <.001) and social class (medium: OR 1.45, CI 95% 1.23-1.71; high: OR 2.00, CI 95% 1.53-2.61; *P* <.001) and eHealth use. Population density was positively associated (cities: OR 1.23, CI 95% 1.02-1.48, *P* <.05). At the country level, only the proportion of elderly people participating in educational activities was significantly associated with eHealth use (OR 1.06, CI 95% 1.01-1.13, *P* =.02).  Gender, employment status, marital status, and household size were not associated with eHealth use. |
|  |  |  | Age  (years) | Mean | | | 73.04 | | | | | | | 71.96 | | | | | | | | 0.97 (0.96-0.98) ^a^ | | | | | | | |  |
|  |  |  | Gender | Male | | | 2676 (46.24) | | | | | | | 536 (48.25) | | | | | | | | Ref | | | | | | | |  |
|  |  |  |  | Female | | | 3111 (53.76) | | | | | | | 576 (51.75) | | | | | | | | 1.02 (0.88-0.19) ^a^ | | | | | | | |  |
|  |  |  | Age when education was completed | <16 | | | 1855 (32.05) | | | | | | | 153 (13.75) | | | | | | | | Ref | | | | | | | |  |
|  |  |  |  | 16-19 | | | 2350 (40.61) | | | | | | | 401 (36.03) | | | | | | | | 1.43 (1.15-2.79) ^a^ | | | | | | | |  |
|  |  |  |  | ≥20 | | | 1582 (27.34) | | | | | | | 559 (50.22) | | | | | | | | 1.95 (1.54-2.46) ^a^ | | | | | | | |  |
|  |  |  | Social class | Low | | | 2868 (49.56) | | | | | | | 354 (31.81) | | | | | | | | Ref | | | | | | | |  |
|  |  |  |  | Medium | | | 2545 (42.98) | | | | | | | 597 (53.64) | | | | | | | | 1.45 (1.23-1.71) ^a^ | | | | | | | |  |
|  |  |  |  | High | | | 374 (6.46) | | | | | | | 162 (14.56) | | | | | | | | 2.00 (1.53-2.61) ^a^ | | | | | | | |  |
|  |  |  | Employment status | Employed | | | 324 (5.60) | | | | | | | 95 (8.54) | | | | | | | | Ref | | | | | | | |  |
|  |  |  |  | Not employed | | | 5463 (94.40) | | | | | | | 1018 (91.46) | | | | | | | | 0.92 (0.70-1.21) | | | | | | | |  |
|  |  |  | Marital status | With partner | | | 3152 (54.47) | | | | | | | 684 (61.46) | | | | | | | | Ref | | | | | | | |  |
|  |  |  |  | Without partner | | | 2635 (45.53) | | | | | | | 429 (38.54) | | | | | | | | 0.81 (0.63-1.03) | | | | | | | |  |
|  |  |  | Household size | One | | | 2246 (38.82) | | | | | | | 366 (32.88) | | | | | | | | Ref | | | | | | | |  |
|  |  |  |  | Two | | | 3024 (52.26) | | | | | | | 673 (60.47) | | | | | | | | 1.17 (0.90-1.51) | | | | | | | |  |
|  |  |  |  | Three | | | 345 (5.96) | | | | | | | 45 (4.04) | | | | | | | | 0.79 (0.521.17) | | | | | | | |  |
|  |  |  |  | Four or more | | | 171 (2.96) | | | | | | | 29 (2.61) | | | | | | | | 1.20 (0.74-1.93) | | | | | | | |  |
|  |  |  | Population density | Rural area | | | 1673 (28.91) | | | | | | | 286 (25.70) | | | | | | | | Ref | | | | | | | |  |
|  |  |  |  | Towns and suburbs | | | 1974 (34.11) | | | | | | | 390 (35.04) | | | | | | | | 1.11 (0.921.34) | | | | | | | |  |
|  |  |  |  | Cities | | | 2140 (36.98) | | | | | | | 437 (39.26) | | | | | | | | 1.23 (1.02-1.48) ^a^ | | | | | | | |  |
| Petersen et al.  (2017)  Denmark  [50] | 1. Prevalence  2. Danish adults  (N = 1,059)  3. Descriptive  4. eHealth portal | Access and use of Sundhed.dk, a national health portal |  | | | | n | | | | | | | Has visited Sundhed.dk  (%) | | | | | | | | Has not visited Sundhed.dk (%) | | | | | | | | People with only primary school education are less likely to use the Danish national health portal than those with higher education. Only 21% of people with only primary school education have used the portal while 60% of people with a high education have used it. |
|  |  |  | Education level | Long higher  (>4years) | | | 218 | | | | | | | 62 | | | | | | | | 32 | | | | | | | |  |
|  |  |  |  | Medium higher  (3-4years) | | | 344 | | | | | | | 60 | | | | | | | | 32 | | | | | | | |  |
|  |  |  |  | Short higher  (<3years) | | | 144 | | | | | | | 57 | | | | | | | | 32 | | | | | | | |  |
|  |  |  |  | Vocational | | | 173 | | | | | | | 49 | | | | | | | | 42 | | | | | | | |  |
|  |  |  |  | High School | | | 100 | | | | | | | 51 | | | | | | | | 27 | | | | | | | |  |
|  |  |  |  | Secondary School | | | 20 | | | | | | | 47 | | | | | | | | 49 | | | | | | | |  |
|  |  |  |  | Primary School | | | 59 | | | | | | | 21 | | | | | | | | 65 | | | | | | | |  |
